# Identification of heat-tolerance QTLs and high-temperature stress-responsive genes through conventional QTL mapping, QTL-seq and RNA-seq in tomato

**DOI:** 10.1186/s12870-019-2008-3

**Published:** 2019-09-11

**Authors:** Junqin Wen, Fangling Jiang, Yiqun Weng, Mintao Sun, Xiaopu Shi, Yanzhao Zhou, Lu Yu, Zhen Wu

**Affiliations:** 1 College of Horticulture, Nanjing Agricultural University, Weigang NO 1, Nanjing, 210095 China; 2 University of Wisconsin-Madison, Madison, USA

**Keywords:** Tomato, QTL-seq, RNA-seq, Heat tolerance

## Abstract

**Background:**

High temperature is one of the major abiotic stresses in tomato and greatly reduces fruit yield and quality. Identifying high-temperature stress-responsive (HSR) genes and breeding heat-tolerant varieties is an effective way to address this issue. However, there are few reports on the fine mapping of heat-tolerance quantitative trait locus (QTL) and the identification of HSR genes in tomato. Here, we applied three heat tolerance-related physiological indexes, namely, relative electrical conductivity (REC), chlorophyll content (CC) and maximum photochemical quantum efficiency (F_v_/F_m_) of PSII (photosystem II), as well as the phenotypic index, the heat injury index (HII), and conventional QTL analysis combined with QTL-seq technology to comprehensively detect heat-tolerance QTLs in tomato seedlings. In addition, we integrated the QTL mapping results with RNA-seq to identify key HSR genes within the major QTLs.

**Results:**

A total of five major QTLs were detected: *qHII-1-1*, *qHII-1-2*, *qHII-1-3*, *qHII-2-1* and *qCC-1-5* (*qREC-1-3*). *qHII-1-1*, *qHII-1-2* and *qHII-1-3* were located, respectively, in the intervals of 1.43, 1.17 and 1.19 Mb on chromosome 1, while the interval of *qHII-2-1* was located in the intervals of 1.87 Mb on chromosome 2. The locations observed with conventional QTL mapping and QTL-seq were consistent. *qCC-1-5* and *qREC-1-3* for CC and REC, respectively, were located at the same position by conventional QTL mapping. Although *qCC-1-5* was not detected in QTL-seq analysis, its phenotypic variation (16.48%) and positive additive effect (0.22) were the highest among all heat tolerance QTLs. To investigate the genes involved in heat tolerance within the major QTLs in tomato, RNA-seq analysis was performed, and four candidate genes (*SlCathB2, SlGST, SlUBC5,* and *SlARG1*) associated with heat tolerance were finally detected within the major QTLs by DEG analysis, qRT-PCR screening and biological function analysis.

**Conclusions:**

In conclusion, this study demonstrated that the combination of conventional QTL mapping, QTL-seq analysis and RNA-seq can rapidly identify candidate genes within major QTLs for a complex trait of interest to replace the fine-mapping process, thus greatly shortening the breeding process and improving breeding efficiency. The results have important applications for the fine mapping and identification of HSR genes and breeding for improved thermotolerance.

**Electronic supplementary material:**

The online version of this article (10.1186/s12870-019-2008-3) contains supplementary material, which is available to authorized users.

## Background

Tomato (*Solanum lycopersicum*) is an important horticultural crop that is thermophilic but cannot withstand high temperatures. The optimum temperatures for growth during the day and night are 25–33 °C and 15–20 °C, respectively [1]. High ambient temperature inhibits tomato growth and development, thus reducing its yield and quality [2]. The heat tolerance of plants is influenced by the environment, management practices and especially genotype [3]. Therefore, the most fundamental way to solve this problem is to identify HSR genes and develop heat-tolerant varieties [4].

Tomato heat tolerance is a quantitative trait, and quantitative trait locus (QTL) mapping is an effective way to identify genes responsible for heat tolerance [5]. The key to QTL analysis of heat tolerance is precise phenotyping [6]. A number of studies have evaluated heat tolerance in tomato using various parameters, such as a phenotypic index [7], physiological and biochemical stress indexes [8, 9], and a microscopic observation index [10]. A phenotypic index is a direct diagnostic tool that can directly reflect the degree of heat damage [11]. For example, the heat injury index (HII) is a preferred index for the degree of heat damage to tomato seedlings under high-temperature stress [12]. Physiological indexes respond faster than morphological changes to high-temperature stress. For example, membrane damage is a primary symptom of heat injury, and heat tolerance is positively correlated with the electrolytic leakage rate; often, relative electrical conductivity (REC) is used to evaluate heat tolerance [13, 14]. High-temperature stress leads to the inhibition of chlorophyll biosynthesis [15]; hence, chlorophyll content (CC) can be used as an effective evaluation index for high-temperature stress. Photosystem II (PSII) is the most thermally labile component of the electron transport chain [16]. The inhibition of photosystem II (PSII) activity under high-temperature stress leads to a decrease in the variable chlorophyll fluorescence, and the maximum photochemical quantum efficiency (F_v_/F_m_) of photosystem II (PSII), as one of the most sensitive chlorophyll fluorescence parameters of plants under high-temperature stress, can be used for heat tolerance evaluation [17]. Therefore, in the present study, we evaluated the heat tolerance of tomato seedlings in a segregating population by using the morphology-based HII and three physiological indexes, namely, REC, CC and F_v_/F_m_.

Some QTLs associated with heat tolerance have been identified in tomato [18–21]. However, due to the use of a small population and an insufficiently dense genetic map, many heat-tolerance QTLs covered a large area with a large number of candidate genes [22]. Thus, fine mapping is needed to identify the major QTLs and key genes involved in heat tolerance, but fine mapping requires the construction of advanced mapping groups and intensive genetic maps, which are labor-intensive and time-consuming [23]. With the rapid development of next-generation sequencing (NGS) technologies, QTL mapping based on high-throughput sequencing has developed rapidly, which has enabled the completion of interval mapping and candidate gene identification through sequencing. More broadly applicable strategies are available for identifying major QTLs controlling grain length and weight [24], early flowering time in cucumber [25] and flowering time in chickpea [26]. These studies showed that QTL-seq is a quick and efficient method for identifying genomic regions harboring the major QTL of the target gene. Although this QTL mapping method is convenient and could narrow down the size of QTL regions, it also has many limitations, such as the sequencing depth and method of biological information analysis, which will affect the accuracy of gene detection, and it is difficult to identify major QTLs explaining a large amount of phenotypic variation, especially QTLs for quantitative agronomic traits, by whole-genome sequencing-based QTL mapping (QTL-seq) [27]. To overcome these limitations, this study intends to combine conventional QTL analysis and QTL-seq technology to detect heat-tolerance QTLs in tomato. The strategy has already been applied to quickly and accurately identify the major QTLs and candidate genes related to flowering time in cucumber [25], 1000-seed weight in chickpea [28] and plant height in soybean [29], but it has not been used to identify QTLs and candidate genes associated with heat tolerance in tomato.

When major heat-tolerance QTLs are obtained, the next step is identifying the functional genes within the QTLs. It is essential for us to extract HSR genes that are beneficial for the elucidation of the high-temperature stress mechanism and for improving tomato heat tolerance by genetic engineering. Conventional fine-mapping methods can be used for target gene identification [30–32]. However, fine mapping mainly depends on recombination events. For regions with low recombination frequency or even completely suppressed recombination, the candidate genes cannot be further identified [33]. Moreover, fine mapping is tedious and time-consuming. Thus, we can use the strategy of QTL mapping combined with RNA-seq instead of fine mapping to rapidly detect the target genes underlying major QTLs. For example, researchers integrated QTL mapping and RNA-seq to identify candidate genes for pod number in rapeseed (*Brassica napus* L.) [34], the gene *PmSGD* related to powdery mildew in wheat [35] and genes controlling capsaicinoid content in *Capsicum chinense* [36], but the application of this method for the exploration of HSR genes in tomato has not been reported.

Based on a previous study with screening of 67 tomato genotypes under heat stress using F_v_/F_m_ [37], the heat-susceptible genotype LA1698 and the heat-tolerant genotype LA2093 were selected to construct a mapping population. We were able to reliably detect overlapping QTLs with high phenotypic variation explained for heat tolerance-related physiological indexes, including REC, CC, and F_v_/F_m_, as well as the phenotypic HII by using conventional QTL mapping and QTL-seq in an F_2_ population derived from a cross between the heat-susceptible genotype LA1698 and the heat-tolerant genotype LA2093. RNA-seq was employed to reveal some key candidate genes responsible for high-temperature stress within the overlapping major QTLs obtained from a comparison and analysis of the results of conventional QTL mapping and QTL-seq in tomato. Then, the key heat-tolerance candidate genes were further screened by qRT-PCR and biological function analysis. Therefore, the main purpose of this study is as follows:

1. to detect major heat-tolerance QTLs in tomato seedlings
2. and to identify high-temperature stress-responsive genes within the major QTLs.

We use QTL mapping and RNA-seq to identify HSR genes and discuss the method of gene fine mapping. This research not only will have a guiding effect on breeding for improved thermotolerance but also lays a theoretical foundation for uncovering the molecular regulatory mechanism of heat tolerance in tomato.

## Results

### Conventional QTL mapping of heat tolerance-related physiological traits in tomato

Relative electrical conductivity (REC), chlorophyll content (CC), and the maximum photochemical quantum efficiency (F_v_/F_m_) of photosystem II (PSII) were measured in parental lines and the F_2_ population. Relevant statistics are presented in Table 1. REC, CC and F_v_/F_m_ displayed significant differences between the two parental lines. After treatment, the REC, CC and F_v_/F_m_ of LA1698 were 0.61, 2.49 (mg/g) and 0.72, respectively. The REC, CC and F_v_/F_m_ of LA2093 were 0.45, 2.98 (mg/g) and 0.77, respectively. The CC and F_v_/F_m_ of LA1698 were significantly lower than those of LA2093, and the REC of LA1698 was significantly higher than that of LA2093. The mean CC and F_v_/F_m_ of the F_2_ population were 2.81 (mg/g) and 0.68, respectively, which were close to the mid-parent values, whereas the mean EC was 0.63, which was higher than that of the heat-sensitive parent. The ranges of the three parameters among the F_2_ plants were beyond those of the two parental lines, suggesting transgressive inheritance of these traits (Table 1). All three traits exhibited continuous and largely normal distributions (Additional file 12: Figure S1), confirming the quantitative nature of this tomato population, which could be used for QTL mapping analysis.

**Table 1:** Basic statistics of three heat tolerance indexes of the parental lines and F_2_ population

Physiological indexes	Parents		F_2_ population				
	LA1698	LA2093	Mean	Range	Stdev.	Skewness	Kurtisos
REC	0.61*	0.45	0.63	0.21–0.81	0.13	−1.59	2.16
CC	2.49	2.98*	2.81	2.08–3.39	0.28	−0.16	− 0.39
F_v_/F_m_	0.72	0.77*	0.68	0.49–0.80	0.06	−0.25	0.04

REC indicates relative electrical conductivity, CC indicates chlorophyll content, F_v_/F_m_ indicates maximum photochemical quantum efficiency. Level of significant differences are shown (* *P* < 0.05)

Among the 516 SSR markers screened, 146 were polymorphic between the two parental lines, with a polymorphism level of 28.25%. Of the 236 InDel markers screened, 67 (28.39%) were polymorphic. Among the 213 polymorphic markers, 137 were successfully mapped with 144 F_2_ plants. Statistical information for the resulting genetic map is presented in Additional file 1: Table S1 and visually illustrated in Additional file 13: Figure S2. The linkage map contained 137 mapped loci in 12 linkage groups corresponding to the 12 tomato chromosomes. The total length of this map was 1503.82 centimorgans (cM), and the mean marker interval was 10.98 cM. Chromosome 8 had the maximum number of markers, with a total of 16; chromosomes 9 and 12 had the minimum number of markers (seven); and the number of markers of the other chromosomes ranged from 9 to 15. In general, the markers were evenly distributed, making them suitable for QTL mapping analysis.

Using the genotypic and phenotypic data collected from the F_2_ population, QTL analysis was performed with the Inclusive Composite Interval Mapping (ICIM) program (*http://www.isbreeding.net*). At a LOD threshold ≥2.5, a total of 12 QTLs were detected in a heat-stressed environment as being associated with physiological traits in tomato (Table 2; Additional file 13: Figure S2). Seven QTLs for REC were identified on chromosomes 1, 2, 3, 9, and 12, accounting for 1.04–6.70% of the observed phenotypic variance. The most significant QTL (*qREC-1-1*) for REC was linked with markers *W299* and *SL20134_408i*, accounting for 6.7% of the phenotypic variation and exhibiting a LOD score of 11.59. *qREC-9-1* and *qREC-12–1* had positive additive gene effects among the seven QTLs for REC, explained 2.14 and 5.70% of the phenotypic variation, respectively, and indicated that the synergistic gene for the two QTLs that improved heat tolerance was derived from the heat-tolerant parent (LA2093). Three QTLs for CC were identified on chromosomes 1 (*qCC-1-4* and *qCC-1-5*) and 2 (*qCC-2-2*). The phenotypic variance explained by the three QTLs was 1.71, 16.48 and 4.86%, respectively. Among them, *qCC-1-5*, flanked by *SSR134* and *C01M86371*, is worth further study, as it explained the most phenotypic variation (16.48%) and had a positive additive effect (0.22) among all detected heat-tolerance QTLs, showing its potential value in breeding applications. F_v_/F_m_ was linked to two QTLs on chromosomes 5 and 12 with the flanking markers *SSR13*, *SSR115*, *SSRD74* and *LeOH301* and explained 6.94 and 8.80% of the phenotypic variation, respectively. The additive effects of *qF*_*v*_*/F*_*m*_*-5-1* and *qF*_*v*_*/F*_*m*_*-12–2* were 0.18 and 0.03, respectively. Among the 12 QTLs detected by conventional QTL mapping, the additive effect of 6 QTLs was positive, and the effects of the remaining QTLs were negative. The positive additive effect indicated that the allele from the heat-tolerant parent (LA2093) contributed to increased heat tolerance.

**Table 2:** Conventional QTL analysis for heat tolerance-related physiological indexes in the F_2_ population

Trait	CH	QTL^a^	Peak Position (cM)^b^	Flanking markers^c^	Location (Mb)^d^	LOD^e^	PVE (%)^f^	Add^g^
REC	1	*qREC-1-1*	52.0	*W299-SL20134_408i*	24.10–80.54	11.59	6.70	−0.13
	1	*qREC-1-2*	71.0	*SL20134_408i-SSR270*	80.54–80.70	10.62	5.81	−0.17
	**1**	***qREC-1-3***	**156.0**	***SSR134 -C01M86371***	**81.64–86.37**	**3.78**	**5.75**	**−0.15**
	2	*qREC-2-1*	47.0	*SSRD69-C02M48329*	43.48–48.33	5.41	5.66	−0.19
	3	*qREC-3-1*	119.0	*C03M68619-C03M70037*	68.62–70.04	3.24	1.04	−0.05
	9	*qREC-9-1*	18.0	*SSRD133-TGS1354*	1.11–4.01	2.60	2.14	0.07
	12	*qREC-12–1*	13.0	*SL10953_259i-SSRD80*	3.79–36.67	9.13	5.70	0.18
CC	1	*qCC-1-4*	118.38	*C01M75064-SSR134*	75.06–81.64	3.70	1.71	−0.06
	**1**	***qCC-1-5***	**160.66**	***SSR134 -C01M86371***	**81.64–86.37**	**3.72**	**16.48**	**0.22**
	2	*qCC-2-2*	0.01	*SSR96-C02M4005*	39.71–40.00	3.52	4.86	0.05
F_v_/F_m_	5	*q F*_*v*_*/F*_*m*_ *− 5-1*	1.51	*SSR13-SSR115*	2.53–2.73	2.50	6.94	0.18
	12	*qF*_*v*_*/F*_*m*_ *− 12–2*	80.29	*SSRD74- LeOH301*	54.37–63.56	3.00	8.80	0.03

REC indicates relative electrical conductivity, CC indicates chlorophyll content, F_v_/F_m_ indicates maximum photochemical quantum efficiency

The consistent QTLs *qCC-1-5* and *qREC-1-3* on chromosome 1 are shown in bold

^a^the QTLs for REC, CC and F_v_/F_m_ are temporarily named ‘*qREC, qCC and qF*_*v*_*/F*_*m*_ + number of chromosome + number of QTL’

^b^The position of heat tolerance-related QTLs

^c^Flanking markers of heat tolerance-related QTLs

^d^The physical position of flanking markers linked with heat tolerance QTLs

^e^Peak LOD value of the QTL

^f^The phenotypic variation explained by the putative QTL

^g^Additive effect. A positive value indicates that the genes derived from heat tolerance parent LA2093 contributed to increased heat tolerance, and the negative value indicates that genes originated from LA1698 reduce the effect on tomato heat tolerance

### Identification of QTLs controlling the heat injury index in tomato using QTL-seq

**Fig. 1:**
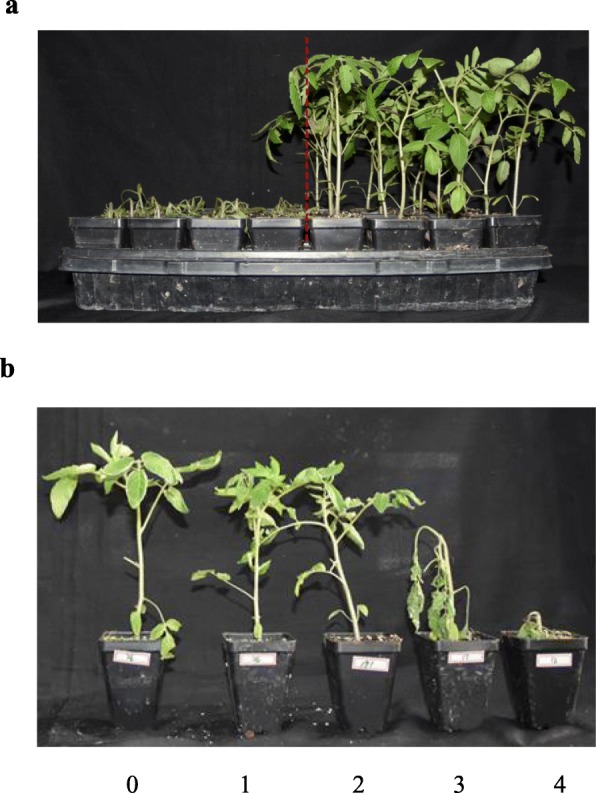
Identification of heat injury index in the F_2_ population. (**a**) The high-quality genomic DNA isolated from leaves of 20 each of the heat-sensitive (the plants at the left of the dotted line) and heat-tolerant (the plants at the right of the dotted line) mapping individuals was pooled at an equal ratio (amount) to constitute an HSB (Heat-sensitive bulk) and HTB (Heat-tolerant bulk) sample, respectively. (**b**) The heat injury degree of the F_2_ population was visually scored with the scale criterion from 0 to 4. The score of 0 meant no obvious heat damage on plants; The score of 1 meant the plant had few leaves wilted and slightly curled at edge; The score of 2 indicated that the plant had 3–4 leaves wilted and badly crimped at edge; The score of 3 represented that the whole plant wilted due to dehydration; The score of 4 described that the plant died

The heat injury phenotypes in the 200 F_2_ plants were found to be significantly different after 48 h of high-temperature stress treatment by observing the growth status of the true leaves and the whole plant. Extremely heat-tolerant strains showed no apparent symptoms of heat injury, and the extremely heat-susceptible strains wilted and died (Fig. 1a). The heat injury degree of the F_2_ population was divided into five levels, and the heat damage of the F_2_ population was visually scored with a scale of 0–4 (Fig. 1b). Continuous variation in the heat injury index of mapping individuals was apparent, consistent with the genetic characteristics of quantitative traits (Additional file 14: Figure S3).

**Fig. 2:**
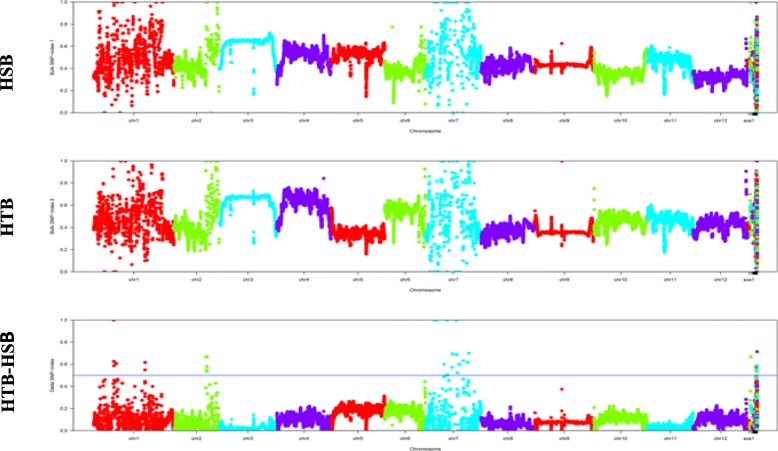
SNP-index graphs of HSB, HTB, and ∆(SNP-index) graphs from QTL-seq analysis. X-axis denotes the position (Mb) of 12 chromosomes of tomato and Y-axis represents the SNP-index of HSB (Heat-sensitive bulk), HTB (Heat-tolerant bulk) and ΔSNP-index of HTB-HSB. SNP-index was estimated based on 1 Mb physical interval with a 10 kb sliding window. Using the statistical confidence intervals under the null hypothesis of no QTL (*P* < 0.01), the Δ(SNP-index) graph was plotted. 12 QTLs were identified on chromosome 1, 2 and 7 (1–2 Mb interval) with the criteria that the SNP-index near to 0 and 1 in HSB and HTB, respectively, and the Δ(SNP-index) was above the confidence value 0.5 (at significance level P < 0.01)

The NGS-based high-throughput sequencing of the two parental genotypes as well as the HSB and HTB resulted in 48,383,616, 42,900,716, 85,307,408 and 83,673,715 high-quality short reads (100 bp in length), respectively, covering 88.60–89.46% of the reference genome. The Best Practices method in GATK was used to detect SNPs [38], and 3,521,275–5,840,665 SNPs were identified in the parental and bulk samples, respectively (Additional file 2: Table S2; Additional file 3: Table S3). The SNP index of each identified SNP differentiating LA1698 and the HSB from LA2093 and the HTB was calculated. The average SNP index across a 1-Mb genomic interval was computed for the HSB and HTB using a 10-kb sliding window and plotted against the 12 tomato chromosomes, and the Δ(SNP index) was then calculated between the two extreme bulk samples (Fig. 2). Following the principle of SNP-index estimation in QTL-seq analysis, 12 genomic regions (the total length was 18.21 Mb) on chromosomes 1, 2 and 7 were identified with a statistical confidence of *P* < 0.01 and threshold value > 0.5000 (Table 3). Three heat-tolerance QTLs were identified on chromosome 1 (*qHII-1-1*, *qHII-1-2*, and *qHII-1-3*), and only one QTL was detected on chromosome 2 (*qHII-2-1*). The largest number of QTLs, eight, was identified on chromosome 7 (10.08 Mb–52.20 Mb). We designated these QTLs *qHII-7-1, qHII-7-2, qHII-7-3, qHII-7-4, qHII-7-5, qHII-7-6, qHII-7-*7 and *qHII-7-8*. All QTLs detected by QTL-seq were in the range of 1–2 Mb and contained a large number of candidate genes; therefore, it was difficult to screen candidate genes directly.

**Table 3:** QTLs for heat injury index detected by QTL-seq

CH	QTL^a^	Chromosome Location(Mb)^b^		Interval (Mb)^c^	SNP-index of HTB^d^		SNP-index of HSB^e^	
		Start	End		Mean	Maximum	Mean	Minimum
**chr1**	***qHII-1-1***	**23.80**	**25.23**	**1.43**	**0.97**	**1**	**0.38**	**0**
**chr1**	***qHII-1-2***	**26.83**	**28.00**	**1.17**	**1**	**1**	**0.40**	**0.29**
**chr1**	***qHII-1-3***	**62.33**	**63.52**	**1.19**	**0.6**	**1**	**0.15**	**0**
**chr2**	***qHII-2-1***	**38.98**	**40.85**	**1.87**	**1**	**1**	**0.42**	**0.33**
chr7	*qHII-7-1*	10.08	11.56	1.48	1	1	0	0
chr7	*qHII-7-2*	12.11	13.49	1.38	1	1	0	0
chr7	*qHII-7-3*	21.94	23.08	1.14	1	1	0.4	0.4
chr7	*qHII-7-4*	24.79	26.90	2.11	0.9	1	0.23	0
chr7	*qHII-7-5*	36.83	38.82	1.99	1	1	0.32	0
chr7	*qHII-7-6*	40.45	42.44	1.99	0.6	0.6	0	0
chr7	*qHII-7-7*	45.75	46.88	1.13	0.86	1	0.23	0
chr7	*qHII-7-8*	50.87	52.20	1.33	1	1	0.32	0

The consistent intervals observed with conventional QTL mapping and QTL-seq are shown in bold

^a^the QTLs for HII are temporarily named ‘*qHII* (Heat injury index) + number of chromosome + number of QTL’

^b^The Physical position of QTLs for HII

^c^The interval (Mb) of QTLs related to HII

^d^The mean and maximum value of SNP-index in HTB

^e^The mean and minimum value of SNP-index in HSB

### Identification of major QTLs controlling heat tolerance

In this study, conventional QTL mapping and new QTL-seq analysis were used to explore heat-tolerance QTLs. Through comprehensive comparison and analysis of the results of the two methods, the major heat-tolerance QTLs and genes were further screened and determined, which could significantly improve the accuracy and effectiveness of major QTL and gene excavation.

**Fig. 3:**
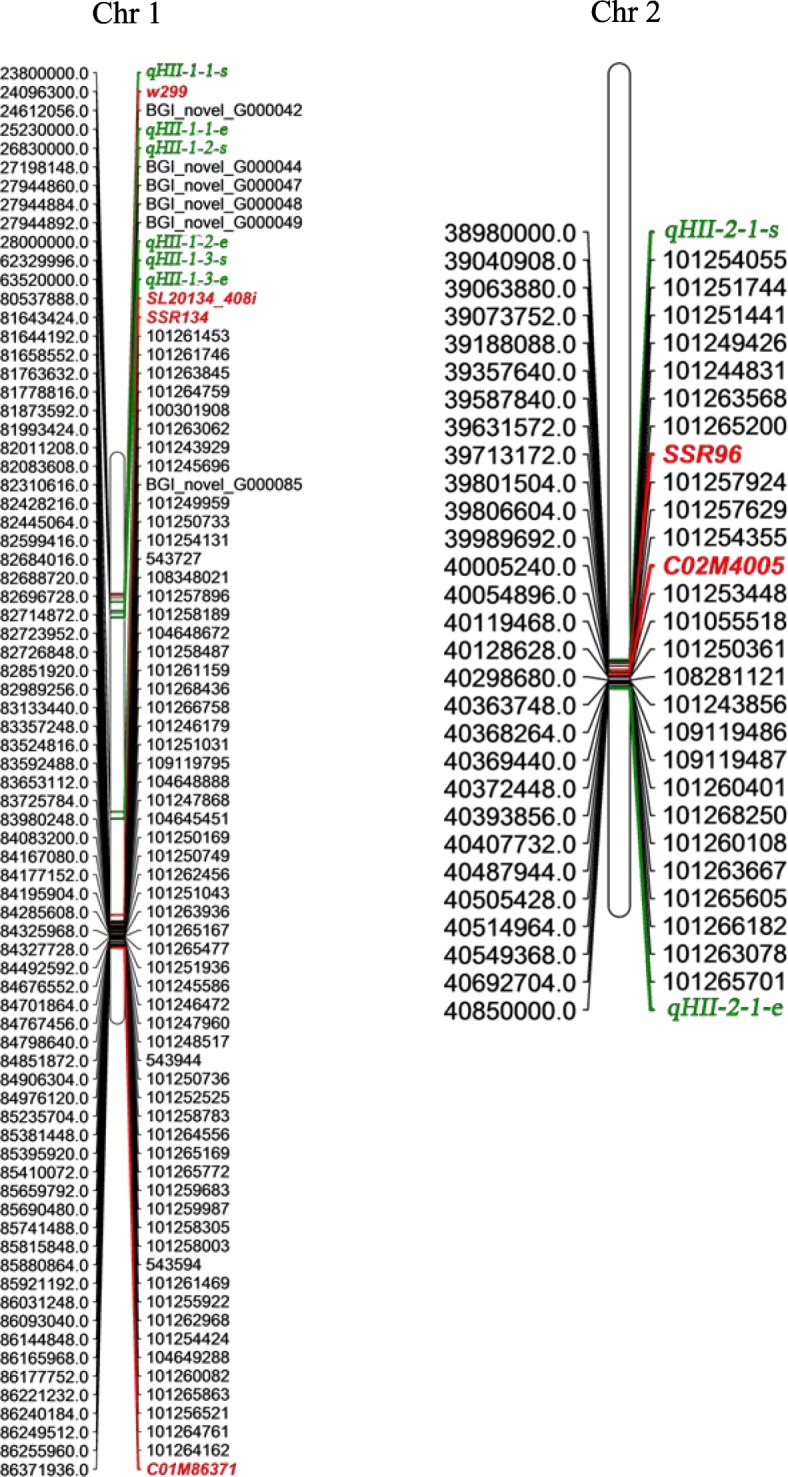
Chromosomal location of genes within the major genomic regions of chromosome 1 and 2. The physical (bp) positions and identity of the genes, markers, QTL intervals mapped on the chromosomes were specified on the left and right side of the chromosomes, respectively. The flanking markers linked with QTLs for heat tolerance that identified by conventional QTL mapping were marked with red italic. The QTL intervals detected by QTL-Seq were represented with green italic ‘QTL-s’ and ‘QTL-e’. 91 DEGs was found in target genomic regions

The heat-tolerance QTL *qREC-1-1*, which was used for conventional QTL analysis, was identified on chromosome 1 with the closest flanking markers *W29*9 and *SL20134_408i*, and it was physically located in the region of 24.10–80.54 Mb. The correspondence of QTL-seq outcomes with conventional QTL mapping results revealed three heat-tolerance QTLs (*qHII-1-1*, *qHII-1-2*, and *qHII-1-3*) on chromosome 1. The mean SNP indexes of *qHII-1-1*, *qHII-1-2*, and *qHII-1-3* were 0.97, 1, and 0.6 in the HTB, respectively, with the highest being 1, while the average SNP indexes in the corresponding region of the HSB were 0.38, 0.40, and 0.15, respectively, with the lowest being 0. The closer the SNP index of the HTB pool was to 1 and the closer the SNP index of the HSB pool was to 0, the higher the confidence of the QTL interval. The physical locations of *qHII-1-1*, *qHII-1-2*, and *qHII-1-3* were 23.80–25.23 Mb, 26.83–28.00 Mb, and 62.33–63.52 Mb, respectively (Table 3); these QTLs were included in the results of conventional QTL analysis (Fig. 3). *qREC-1-1*, which was the most significant QTL, had the highest LOD value among all the heat-tolerance QTLs detected by conventional QTL mapping (11.59).

*qCC-2-2* was identified on chromosome 2 with the flanking markers *SSR96* and *C02M4005* by conventional QTL analysis. The LOD value and phenotypic variation explained were 3.52 and 4.86%, respectively, and the corresponding physical location was 39.71–40.00 Mb. This QTL mapping result was consistent with the result of QTL-seq analysis supporting a major QTL locus, *qHII-2-1*, for heat tolerance in the genomic region of 38.98–40.85 Mb on chromosome 2 (Fig. 3). In this region, the SNP index of the three InDel variations in the HTB pool was 1, and the average SNP index and the lowest SNP index in the HSB pool were 0.42 and 0.33, respectively (Table 3).

Based on the above results, *qHII-1-1*, *qHII-1-2*, *qHII-1-3* and *qHII-2-1*, which were detected by QTL-seq analysis, were also included in the results from conventional QTL mapping and nearly coincided with the conventional mapping results. In addition, in the QTL-seq analysis, the SNP index of these QTLs was close to 1 in the HTB pool and close to 0 in the HSB pool, and the LOD values in the conventional QTL analysis were higher. Therefore, these consistent QTLs have high credibility. Eight heat-tolerance QTLs were also detected on chromosome 7 by QTL-seq analysis, but they were not detected in conventional QTL mapping and thus were not considered.

In addition, *qREC-1-3* and *qCC-1-5*, which were mapped to the same region (81.64–86.37 Mb) on chromosome 1 with the flanking markers *SSR134-C01M86371* and *qCC-1-5*, explained the most phenotypic variation (16.48%) and had a positive additive effect (0.22) among all the heat-tolerance QTLs detected by conventional QTL analysis, showing that the QTL was closely related to heat tolerance and is worthy of gene mining. These findings from the comparison of conventional QTL mapping and QTL-seq confirmed the presence of the major QTLs regulating the heat tolerance of tomato: *qHII-1-1*, *qHII-1-2*, *qHII-1-3*, *qHII-2-*1 and *qCC-1-5* (*qREC-1-3*).

### Transcriptome profiling and analysis of HSR genes

To investigate the genes involved in heat tolerance in tomato, samples collected at 0 h (those kept under normal conditions that were used as the control) and 4 h of 40 °C high-temperature stress were subjected to RNA-seq analysis using the BGISEQ-500 sequencing platform. The sequencing generated 6.79 Gb of data on average, and the average ratio between the sample and reference genomes was 93.66% (Additional file 4: Table S4). A total of 23,458 expressed genes were detected, including 22,612 known genes and 846 predicted novel genes. A total of 14,639 novel transcripts were identified, 11,739 of which belonged to a novel isoform, 857 of which belonged to a novel protein-coding transcript and the remaining 2077 of which belonged to long noncoding RNA (Additional file 5: Table S5).

**Fig. 4:**
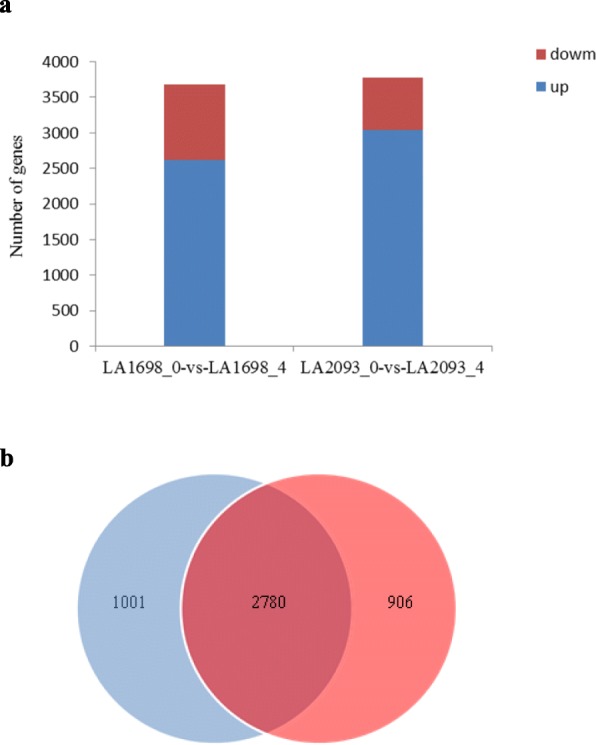
(**a**) Statistics of expressed genes from LA1698_0-VS-LA1698_4 and LA2093_0-VS-LA2093_4. Red and blue pillars represent the significant down and up DEGs with fold change ≥2 or ≤ − 2 and *P*-value ≤0.001, respectively. (**b**) Venn diagram representing the numbers of DEGs from LA1698_0-VS-LA1698_4 and LA2093_0-VS-LA2093_4. 2780 genes were coexpressed in both parents, 906 genes were expressed only in the heat-sensitive parent LA1698, and 1001 genes were expressed only in the heat-tolerant genotype LA2093

After 4 h of high-temperature stress, 3686 and 3781 DEGs were identified in LA1698 and LA2093, respectively (fold change ≥2 or ≤ − 2, *P*-value ≤0.001). For LA1698, 2616 genes were upregulated, and 1070 genes were downregulated, and for LA2093, there were 3030 upregulated genes and 751 downregulated genes (Fig. 4a). In total, 2780 genes were coexpressed in both parents, 906 genes were expressed only in the heat-sensitive parent LA1698, and 1001 genes were expressed only in the heat-tolerant genotype LA2093 (Fig. 4b). Differentially expressed genes are shown in Additional file 6: Table S6, Additional file 7: Table S7 and Additional file 8: Table S8.

To verify the results of RNA sequencing, 2 downregulated and 6 upregulated heat-tolerance genes within major QTLs as well as 4 downregulated DEGs were selected for qRT-PCR analysis between the two parents. The *actin* gene was used as an internal control. As shown in Additional file 15: Figure S4, the results verified by qRT-PCR were consistent with the up- and downregulation trends of the RNA-seq results, indicating that the results from RNA-seq were reliable.

**Fig. 5:**
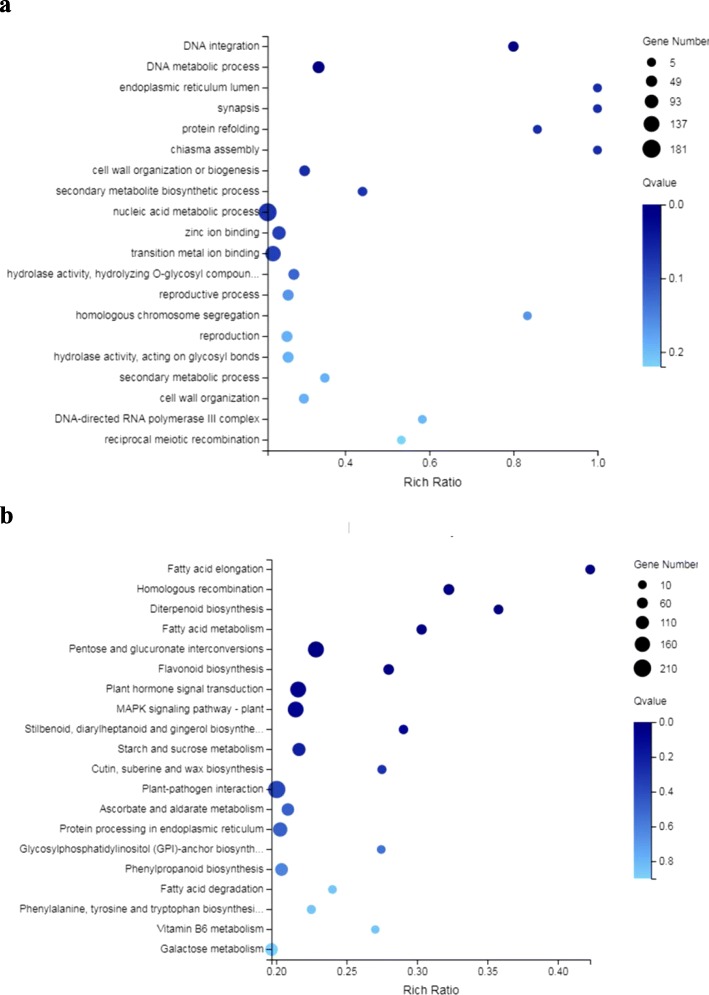
GO (**a**) and KEGG (**b**) enrichment of DEGs. The bubble diagram shows the degree of enrichment of GO and KEGG terms in three categories. By default, the top 20 GO terms with the lowest Q-values were used in the diagram. The X-axis represents the enrichment ratio, and the Y-axis denotes the GO term or KEGG pathway. The size of bubbles indicates the number of genes annotated to a certain GO term or KEGG pathway, and the color represents the Q-value, where the darker the color is, the smaller the Q-value is

The differentially expressed genes (DEGs) were functionally annotated to analyze their gene ontologies (GO) and were also mapped to Kyoto Encyclopedia of Genes and Genomes (KEGG) pathways to assess their functional enrichment. GO functional annotations indicated that the DEGs were involved in a series of biological processes, as follows: *biological regulation*, *cellular process*, *metabolic process* and *response to stimulus*. DEGs were also components of *cells*, *organelles* and *membranes*. In addition, DEGs also had the following activities: *transcription regulation*, *catalytic*, *binding* and *transporter* (Additional file 16: Figure S5a). GO enrichment analysis showed that the DEGs were mainly involved in *DNA integration*, *DNA metabolic process*, *nucleic acid metabolic process*, *zinc ion binding* and *transition metal ion binding under high-temperature stress* (Fig. 5a). Analysis of the KEGG pathways showed that the major pathways involving DEGs were as follows: *signal transduction*, *replication and repair*, *carbohydrate metabolism*, and *environmental adaptation* (Additional file 16: Figure S5b). KEGG enrichment analysis showed that the processes of *pentose and glucuronidate interconversions* and *plant hormone signal transduction*, *the mitogen-activated protein kinases (MAPK) signaling pathway*, *starch and sucrose metabolism*, and *fatty acid metabolism* were significantly enriched under high-temperature stress (Fig. 5b).

### Identification of candidate genes within the major QTLs

**Fig. 6:**
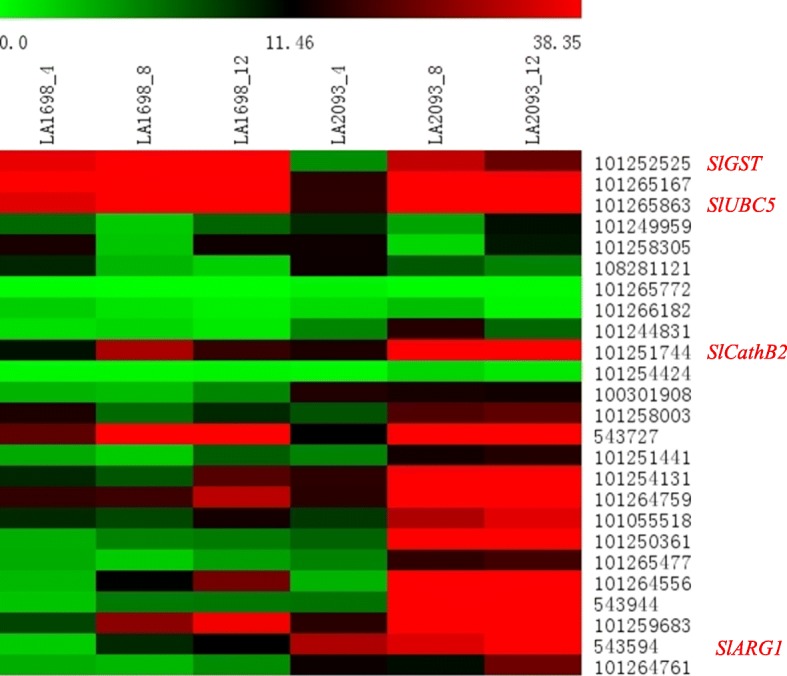
The heat map analysis of the expression of 25 DEGs underlying the major QTLs. The average log signal expression values of genes in various times and parents were denoted at the top with a color scale, in which green, black and red color indicated the low, medium and high level of expression, respectively. The samples and genes used for expression profiling were indicated on the top and right side of the heat map. The expression level of 0 h of parents was used as control and *actin* was used as the endogenous control in qRT-PCR analysis. The expression level of genes calculated by three independent biological replicates with three technical replicates in qRT-PCR assay. Twelve candidate genes showing pronounced differential expression in parents during high-temperature stress were detected. Four genes (Gene ID numbers were: *101252525*, *101,265,863*, *101,251,744*, and *543,944*) were further screened from the 12 candidate genes by combining GO and KEGG function analysis: *SlGST*, *SlUBC5*, *SlCathB2*, and *SlARG1*

A comparison of the results from conventional QTL mapping and QTL-seq analysis combined with RNA-seq analysis was employed to reveal candidate genes regulating the heat tolerance of tomato, and a total of 91 genes were found in the major QTLs *qHII-1-1*, *qHII-1-2*, *qHII-1-3*, *qHII-2-1* and *qCC-1-5* (*qREC-1-3*) (Additional file 9: Table S9). Gene localization to chromosomes 1 and 2 was performed for the distribution of DEGs within major QTL regions using MapChart 2.3 software (Fig. 3). The largest number of DEGs, 43, was found in *qCC-1-5,* and only one differentially expressed gene was found in *qHII-1-1*. Then, 25 genes with high expression levels were selected (the selection criteria were a mean expression of samples with 3 replicates > 5) among the 91 DEGs for further screening of candidate genes by qRT-PCR analysis. The RNA isolated from samples of LA1698 and LA2093 after 4, 8 and 12 h of 40 °C high-temperature treatment (plants kept under normal conditions were used as the control) was amplified with 25 gene-based primers using qRT-PCR analysis. The specific primers were obtained from the database *(**http://biodb.swu.edu.cn/qprimerdb*) provided by Lu et al. [39], and the primer sequences are shown in Additional file 10: Table S10. The results showed that the expression levels of most genes in the heat-tolerant LA2093 genotype were higher than those in the heat-sensitive LA1698 genotype, and the expression levels of the genes at 8 h and 12 h were generally higher than those at 4 h (Fig. 6), indicating that gene expression increased over time with high-temperature treatment. According to the significant difference test of 25 genes from the qRT-PCR analysis (Additional file 17: Figure S6), genes with differential expression in the two parents at 4, 8 and 12 h after high-temperature treatment were screened, and the genes with differential expression during at least two time points were then selected as candidate genes for subsequent biological function analysis in the two parents. Twelve candidate genes were detected by screening. Four genes were further screened from the 12 candidate genes by combining GO and KEGG function analysis (Additional file 11: Table S11): *cathepsin B-like protease 2* (*SlCathB2*), *glutathione S-transferase zeta class-like isoform X1* (*SlGST*), *ubiquitin-conjugating enzyme E2–23 kDa* (*SlUBC5*) and *arginase 1* (*SlARG1*). The corresponding gene ID numbers were *101,251,744*, *101,252,525*, *101,265,863*, and *543,944*, respectively.

## Discussion

### Comprehensive evaluation of the thermotolerance of tomato seedlings through multiple physiological indexes related to heat tolerance

High-temperature stress can elicit a series of physiological and biochemical responses [40, 41]. For example, under a high-temperature stress of 40 °C, the REC of leaves increases. The REC of the heat-sensitive line LA1698 increased more than that of the heat-tolerant line LA2093. Changes in photosynthetic parameters under high-temperature stress are important indexes of thermotolerance. Photochemical responses in the thylakoid lamellae and the metabolism of carbon in the chloroplast stroma have been shown to be the initial responses to high-temperature stress. Thus, REC, CC and F_v_/F_m_ can effectively reflect the degree of damage to the cell membrane, the chloroplast, and PSII during high-temperature stress. In this study, three physiological indexes closely related to plant heat tolerance (REC, CC, and F_v_/F_m_) were used to measure the heat tolerance of the parents and the F_2_ population. The results showed that the REC, CC and F_v_/F_m_ were significantly different between the two parents after 24 h of high-temperature treatment and displayed approximately continuous and wide variation among individuals in the F_2_ population, consistent with expectations for quantitative traits. These results suggested that REC, CC and F_v_/F_m_ can effectively reflect the heat tolerance of tomato varieties under artificially simulated high stress. Compared with natural high temperatures in the field, the microenvironment of artificially simulated high-temperature stress can be precisely controlled, and the character identification with the latter method is more accurate, making it suitable for high-temperature treatment at the seedling stage. Previous studies also showed that the results of REC, CC and F_v_/F_m_ evaluation under artificially simulated high-temperature stress were similar to those under naturally high summer temperatures in the field [14, 42].

Three physiological indexes (REC, CC and F_v_/F_m_) were applied to map heat-tolerance QTLs. Among them, seven QTLs, including QTLs associated with REC (*qREC-1-1, qREC-1-2, qREC-1-3,* and *qREC-2-1*) and CC (*qCC-1-4, qCC-1-5,* and *qCC-2-2*), were colocalized on chromosomes 1 and 2. Both REC and F_v_/F_m_ were associated with one QTL on chromosome 12. The consistent QTL on chromosome 1, *qCC-1-5* (*qREC-1-3*), explained the most phenotypic variation (16.48%) and had a positive additive effect (0.22) among all the detected heat-tolerance QTLs, indicating that the comprehensive application of multiple indicators may be beneficial for detecting important QTLs [20]. QTLs for one index may be small in effect, but combined with other indexes, the effect of the QTLs may become more significant, and genes underlying such QTLs possibly exert pleiotropic effects. These important QTLs or genes can be used quickly and efficiently in breeding, consequently shortening the breeding process. The three heat tolerance-related physiological indexes used in the study represent different physiological processes; however, consistent QTLs were detected for the three indicators, which could be due to pleiotropy or a result of the positive correlations among indicators. These physiological indicators may have the same or similar key regulatory genes or pathways that respond to high-temperature stress. Previous studies examined a large number of QTLs for heat tolerance in tomato and other crops using seedling physiological indicators*.* Xu et al. [19] obtained an SSR and a RAPD marker associated with tomato heat tolerance by measuring plasma membrane permeability, which were located in linkage groups (LGs) 3 and 7, respectively. Talukder et al. [43] located eight QTLs in wheat using CC after thermal stress. Bhusal et al. [44] used chlorophyll content and chlorophyll fluorescence parameters to identify 17 and 23 QTLs related to wheat heat tolerance in a recombinant inbred line (RIL) population, respectively. These results indicated that it is feasible to map QTLs using physiological indexes related to target traits. Most traits, including heat tolerance, are complex quantitative traits controlled by multiple genes and are affected by many factors. The limitation of using a single index to evaluate quantitative traits is major; therefore, the use of multiple appropriate physiological indicators contributes to comprehensive identification of heat-tolerance traits and QTLs.

### Conventional QTL mapping combined with QTL-seq for efficiently and rapidly identifying major QTLs related to heat tolerance

The high-throughput genome-wide QTL-seq strategy has been applied for the identification of major QTLs in a variety of crops, but its application in heat-tolerance QTL mapping in tomato has not been reported. Compared with the conventional fine-mapping process, employing QTL-seq to detect major QTLs of target traits is less time-consuming and requires less effort. However, it is difficult to identify major QTLs explaining large amounts of phenotypic variation, especially QTLs for quantitative agronomic traits, by QTL-seq [27]. Therefore, to efficiently and accurately detect the QTLs related to heat tolerance in the tomato seedling stage, this study employed conventional QTL mapping and QTL-seq to identify major QTLs for heat tolerance in tomato with an F_2_ mapping population. A total of 24 heat-tolerance QTLs were found on chromosomes 1, 2, 3, 5, 7, 9 and 12. Grilli et al. [18] and Xu et al. [21] also detected heat-tolerance QTLs on chromosomes 1 and 7 in tomato, respectively. Xu et al. [19] and Lin et al. [20] also identified some other QTLs responsible for heat tolerance in tomato, but the identified markers and linkage groups were not anchored to chromosomes, hampering wider use of the findings. In our study, *qHII-1-1* (23.8–25.23 Mb), *qHII-1-2* (26.83–28.00 Mb) and *qHII-1-3* (62.33–63.52 Mb), which were identified by QTL-seq, were included in the *qREC-1-1* (24.10–80.54 Mb) QTL detected by conventional QTL mapping. In other words, one QTL with a large region detected by conventional QTL mapping was subdivided into three smaller QTLs using QTL-seq technology. The genomic interval of the QTLs identified by QTL-seq was 1–2 Mb. This reflects the potential advantage of QTL-seq over conventional QTL mapping for high-resolution genome mapping and subsequent fine mapping of target candidate genomic regions harboring major trait-associated QTLs. Our QTL-seq analysis detected a QTL, *qHII-2-1*, ranging from 38.98 to 40.85 Mb on chromosome 2 that overlapped with the QTL *qCC-2-2* (39.71–40.00 Mb) identified in the conventional QTL analysis. In the conventional QTL analysis, *qREC-1-3* and *qCC-1-5* were mapped to the same region (*SSR134-C01M86371*) on chromosome 1, and *qCC-1-5* explained the most phenotypic variation (16.48%) and had a positive additive effect (0.22) among all the detected heat-tolerance QTLs. These overlapping QTLs may represent one or more major heat-tolerance QTLs, which have potential applications. This indicates the consistency between the conventional QTL mapping and QTL-seq methods. Of course, some QTLs detected by the two QTL mapping methods were located in different regions. For example, eight QTLs were revealed on chromosome 7 by QTL-seq but were not detected by conventional QTL mapping. Similarly, five heat tolerance-related QTLs were detected on chromosomes 3, 5, 9 and 12, but this result was not observed in the QTL-seq analysis. This difference may result not only because the two methods have specificity and complementarity but also from the difference between the use of physiological indicators and comprehensive morphological indicators in QTL mapping. Through the combination of phenotypic and physiological indexes, conventional QTL analysis and the new QTL-seq strategy, a total of five major QTLs were detected: *qHII-1-1, qHII-1-2, qHII-1-3*, *qHII-2-1* and *qCC-1-5* (*qREC-1-3*). *qHII-1-1*, *qHII-1-2* and *qHII-1-3* were located, respectively, in the intervals of 1.43, 1.17 and 1.19 Mb on chromosome 1, while the interval of *qHII-2-1* was located in the intervals of 1.87 Mb on chromosome 2. The locations were consistent between conventional QTL mapping and QTL-seq. Although *qCC-1-5* (*qREC-1-3*) was not detected in the QTL-seq analysis, *qCC-1-5* and *qREC-1-3* were located at the same position based on different physiological indicators in conventional QTL mapping; the phenotypic variation explained (16.48%) and positive additive effect (0.22) of *qCC-1-5* were the highest among all the heat-tolerance QTLs identified by conventional analysis, showing that the strategy of conventional QTL mapping combined with QTL-seq technology can quickly and efficiently detect major QTL-related traits, especially QTLs for quantitative traits with a complex genetic basis. The two mapping methods can complement and verify each other and fully explore the QTLs of target traits.

### RNA-seq identified candidate genes within the major QTLs

Although the strategy of QTL mapping combined with RNA sequencing has been applied in the identification of related genes in rapeseed, wheat, pepper and other crops [34–36], the application of this strategy for revealing tomato HSR genes has not been reported. To identify candidate genes within the major QTL regions, we performed RNA-seq in LA1698 and LA2093, revealing some key genes that might be involved in heat tolerance in tomato. Compared with the heat-sensitive genotype LA1698, the heat-tolerant parent LA2093 had more upregulated genes and fewer downregulated genes, showing that the response of the heat-tolerant genotype to high-temperature stress was mainly based on the positive regulation of genes, while the response of the heat-sensitive genotype to high-temperature stress was based on the downregulation of genes. A total of 2780 coexpressed genes were identified in the parents, reflecting the common HSR genes of cultivated and gooseberry tomatoes. The number of specific heat-tolerance genes in currant tomato was higher than that in cultivated tomato, indicating that wild currant tomato had more HSR genes than cultivated tomato, and these genes may partly explain the molecular differences in heat tolerance between currant tomato and cultivated tomato. KEGG enrichment analysis showed that the processes of *pentose and glucuronidate interconversions* and *plant hormone signal transduction*, *the MAPK signaling pathway*, *starch and sucrose metabolism*, and *fatty acid metabolism* were significantly enriched under high-temperature stress, which has been confirmed to be related to the high-temperature stress response under high temperature, and *plant hormone signal transduction* and the *MAPK signaling pathway* are related to high-temperature stress signaling [45–48].

Our experiments identified 25 DEGs with high expression levels in major heat-tolerance regions by RNA-seq analysis, and 25 DEGs were further screened by qRT-PCR and biological function analysis. Finally, four candidate genes related to heat tolerance were found, namely, *SlCathB2*, *SlGST*, *SlUBC5* and *SlARG1*. *SlCathB2* was located in *qHII-2-1*, while the remaining three genes were identified in *qCC-1-5*, indicating that the combination of QTL mapping and RNA-seq is more conducive to rapidly identifying candidate genes related to target traits within major QTLs than conventional fine-mapping processes, which provides a rapid approach for mapping target genes. In addition, a previous study revealed that using this strategy can generate a high-resolution genetic map [49]. Identification of candidate genes within major QTLs is beneficial for rapidly improving the heat tolerance of crops by genetic engineering and marker-assisted selection (MAS)-based breeding using flanking markers of major QTLs.

Four HSR candidate genes were identified in this study, and *SlCathB2* (*cathepsin B-like protease 2*) belongs to the C1A family of peptidases and has the activity of peptidases. Currently, most studies on *SlCathB2* are performed in animals, and three *CathB* homologous genes *(AtCathB1*, *AtCathB2* and *AtCathB3*) have been identified in the model plant *Arabidopsis thaliana* [50]. Cai et al. [51] indicated that the downregulation of *AtCathB2* reduced reactive oxygen species (ROS) accumulation and ER stress-induced PCD (ERSID) in response to abiotic stress. *SlGST* (glutathione S-transferase zeta class-like isoform X1), belonging to the glutathione transferase family, has been found to play an important role in reducing the toxicity of foreign substances, transmembrane transport, protecting cells from oxidative damage, maintaining metabolism and responding to a variety of abiotic stresses [52]. When plants are subjected to stresses, such as high salt, drought, and heavy metals, the expression of *GST* is enhanced, which can effectively remove reactive oxygen species and protect the cell membrane structure and protein activity of plants [53]. *SlUBC5* (*ubiquitin-conjugating enzyme E2–23 kDa*) is part of the ubiquitin ligase superfamily and has the function of transferring ubiquitin molecules to the target protein. Ubiquitination has a significant effect on the biological processes of DNA damage repair, transcriptional regulation and cell death [54]. *Ubiquitin binding enzyme E2* plays an important role in response to biological stress [55], osmotic stress [56], drought stress [57] and salt stress [58]. *SlARG1* (*arginase 1*) is classified in the urease gene family and has special sites that bind to Mn ions. To date, most studies have been conducted on the relationship between *arginase* and plant stress responses, and most of them have focused on the regulation of polyamines and nitric oxide (NO) metabolic pathways [59]. Polyamines and NO act as signaling molecules to regulate plant development and a series of biological and abiotic stress responses [60, 61], and *arginase* can regulate the upstream reactions of the polyamine and NO metabolic pathways to control the polyamine and NO contents in plants and indirectly regulate plant development and response to biological and abiotic stresses [62]. Siddappa et al. [63] found that the arginine content of coriander significantly increased in response to biological and abiotic stresses.

## Conclusions

In conclusion, this study demonstrated that the combination of conventional QTL mapping and QTL-seq analysis using different physiological and phenotypic indexes can quickly, efficiently and accurately detect the major QTLs for target traits and that integrating QTL mapping with RNA-seq data enables the rapid identification of candidate genes within major QTLs for a complex trait of interest. Using conventional QTL mapping, QTL-seq analysis, and RNA-seq, a strategy of QTL mapping and candidate gene identification can replace the process of fine mapping, thus greatly simplifying the process of QTL mapping and target gene identification, laying a foundation for shortening the breeding process and improving breeding efficiency. In this study, 5 major QTLs controlling heat tolerance in tomato were detected for the first time by integrating conventional QTL mapping and QTL-seq analysis, and by combining these methods with RNA-seq, 4 candidate genes (*SlCathB2, SlGST, SlUBC5,* and *SlARG1*) responsible for heat tolerance were identified within the major QTLs. Once functionally validated, the candidate genes can be utilized as potential candidates for marker-assisted genetic improvement of tomato to enhance heat tolerance as well as yield.

## Methods

### Plant materials and construction of mapping populations

Based on a previous study with screening of 67 tomato genotypes under high-temperature stress using F_v_/F_m_ [37], the heat-susceptible cultivated tomato (*Lycopersicon esculentum*) genotype LA1698 and the heat-tolerant currant tomato (*Lycopersicon pimpinellifolium*) genotype LA2093, with contrasting high-temperature stress responses, were used in this study. Seeds of both lines used in this work were obtained from the Tomato Genetic Resource Center (TGRC, University of California, CA, US). An F_2_ population was developed from a single self-pollinated F_1_ plant from a cross between LA1698 (maternal parent) and LA2093 (paternal parent). The populations used in the conventional QTL mapping and QTL-seq analysis consisted of 144 and 200 F_2_ seedlings, respectively.

### Conventional QTL mapping

Conventional QTL mapping was used to determine the number and locations of QTLs for heat tolerance-related physiological traits in tomato. Seeds of the two parental lines and the F_1_ and F_2_ populations were used for the present study. Prior to germination, the seeds were treated with hot water at 50–55 °C for 30 min in a water bath and stirred constantly to reduce viral contamination. The seeds were soaked for 3 h after the water cooled, washed three times with deionized water and then placed into petri dishes with 20 mL of deionized water for 7–8 h. Subsequently, the seeds were germinated at 28 °C in a growth chamber. After germination, the seeds were sown in 72-cell plugs with a commercial organic culture medium (perlite:vermiculite:turfy soil = 1:1:2, Zhenjiang Xingnong Organic Fertilizer Company, China) and placed in a light incubator (Dongnan Instrument, RDN-560E-4, Ningbo, China) with a 25 °C day/18 °C night temperature, 12 h/12 h (day/night) photoperiod, 360 μmol m^− 2^ s^− 1^ photosynthetically active radiation and 75% relative humidity. Fifteen days after planting, parental, F_1_ (12 plants each) and individual F_2_ seedling plants with uniform growth (total: 144) were transplanted into nutrient bowls (11 cm diameter × 9 cm height) filled with the same commercial organic culture medium. Transplanted seedlings were kept in the same growth chamber under the same conditions. When the seedlings had five true leaves and one heart leaf (12 days after transplanting), the temperature of the growth chamber was raised to 40 °C day/40 °C night, while the other parameters remained the same. After a treatment of 24 h, leaf samples from each plant were collected for analysis of relative electrical conductivity (REC), chlorophyll content (CC) and the maximum photochemical quantum efficiency (F_v_/F_m_) of PSII. All the parameters of the parents and F_1_ plants were determined in three biological replicates, with four seedlings in each replicate; each index of the F_2_ population was determined for a single plant, and each plant was measured three times. For each parameter, data from three replicates were collected, and the mean was used for QTL analysis. The second, third and fourth leaves counted from the top location of each plant were excised for determination of REC, CC and F_v_/F_m._ The determination of relative conductivity was based on the method of Washburn [64], the chlorophyll content was determined following Haboudane et al. [65], and the measurement of F_v_/F_m_ followed the protocol of Bredahl et al. [66].

Genomic DNA of all tested seedlings was extracted from the tissues of the young leaves near the top of the seedlings using a DNA extraction kit (Takara, Shanghai, China). The SSR and InDel primers, which were synthesized commercially by Sipujing Biological Corporation (Beijing, China), were selected from the Sol Genomics Network (*SGN,* *http://solgenomics.net/*) database, the Tomato SBM and Marker Database (*http://www.kazusa.or.jp/tomato/*) or previous publications [67, 68]. Construction of the genetic map and QTL mapping were conducted by QTL IciMapping 3.0. Using the genotypic and phenotypic data collected from the F_2_ population, QTL analysis was performed with the ICIM (Inclusive Composite Interval Mapping) program (*http://www.isbreeding.net*), with a LOD threshold of ≥2.5.

### QTL-seq analysis

In this experiment, QTL-seq technology was used to detect QTLs for the heat injury index. The individuals and parental genotypes used for mapping and for identification of the HII were cultivated in the same way as described above. When the seedlings reached the stage of five true leaves and one heart leaf (12 days after transplanting), 200 F_2_ individuals along with the parental genotypes with the same growth status were selected, F_2_ individuals were numbered from 1 to 200, and the fourth leaf from the top of each plant was collected and stored in a − 80 °C ultra-low-temperature refrigerator for subsequent parent and extreme pool QTL-seq analysis. Then, the temperature of the growth chamber was raised to 40 °C day/40 °C night, while the other parameters remained the same. The HII was measured after 48 h of treatment. Determination of the HII followed the method of Yin et al. [69].

For the QTL-seq study, 20 each of the heat-tolerant and heat-sensitive mapping individuals, representing the two extreme ends of the HII normal frequency distribution curve, were screened based on the single-plant selection rate, assuming that 10% of the population showed extreme characters. Then, leaf samples preserved before heat treatment were selected according to the corresponding numbers. Leaf samples of 20 extremely heat-tolerant and 20 extremely heat-sensitive F_2_ single strains as well as the parents were sent to Meiji Biological Company (Shanghai, China, *http://www.majorbio.com/*) for QTL-seq analysis, where the sequencing depth of the parents was 10x and that of the two extreme pools was 20x. The high-quality genomic DNA isolated from leaves of 20 each of the heat-tolerant and heat-sensitive mapping individuals was pooled at an equal ratio (amount) to constitute an HTB (heat-tolerant bulk) sample and an HSB (heat-sensitive bulk) sample, respectively. Illumina PE150 sequencing libraries (read length: 300 bp) were constructed and sequenced individually using the Illumina HiSeq™ platform. The raw data with a Q-score of 30 across > 95% of samples were considered high quality. The filtered high-quality clean data obtained from the two bulks and the parental genotypes were aligned to the *SL2.50* (*https://www.ncbi.nlm.nih.gov/assembly/GCF_000188115.3*) reference genome using the Burrows-Wheeler alignment (BWA) tool [70]. Then, we obtained the location attributions of the sequence, namely, the BAM file. The GATK Best Practices process [38] was used to revise the BAM file and detect the SNP and small InDel markers. Molecular markers with a read depth > 2x were selected for BSA correlation analysis, and sliding-window methods were used to calculate the SNP index of the whole genome in order to eliminate false-positive QTLs [71].

The SNP index and ∆(SNP index), following the parameters recommended by Illa et al. [72], were used to identify candidate genomic regions associated with heat tolerance in tomato. The SNP index is the proportion of reads harboring the SNP that are different from the reference sequence, and the Δ(SNP index) is obtained by calculating the SNP index difference between the HTB and HSB samples. The SNP index were measured as “0” or “1” based on the entire short reads containing genomic fragments derived from LA1698 and LA2093. An average ΔSNP index of SNPs mapped across the 12 tomato chromosomes was calculated using sliding-window analysis with a 1-Mb window size and 10-kb increment. The SNP-index graphs for the HTB pool and HSB pool, as well as the corresponding Δ(SNP index) graph, were plotted to generate SNP index plots. We calculated the statistical confidence intervals of the Δ(SNP index) with a 99% read depth under the null hypothesis of no QTLs.

### RNA-seq analysis

To identify the HSR genes of tomato under high-temperature stress, RNA-seq analysis was performed using heat-sensitive (LA1698) and heat-tolerant (LA2093) plants. The parental genotypes were cultivated in the same way as above until five true leaves and one heart leaf were present (12 days after transplanting). Plants with uniform growth (the seedlings had five true leaves and one heart leaf) were selected for the 40 °C day/40 °C night high-temperature stress treatment, while the other parameters remained the same. The second and third leaves counted from the top location of each plant were quickly collected and stored at − 80 °C after 0, 1, 4, 8, 12 and 24 h of high-temperature treatment. The second leaf was used to identify the optimum time point for transcriptome sequencing by determining the heat-related physiological indexes, and the third leaf was used for RNA-seq and real-time quantitative PCR (qRT-PCR). Three biological replicates were used for each time point. To reduce the sampling error caused by individual differences, five mixed samples were taken for each replicate. The same plants were not used for repeated sampling. Through determination and analysis of the physiological indexes related to heat tolerance at each time point of the two parents (Additional file 18: Figure S7), we found that ascorbic acid reductase (APX), glutathione reductase (GR), peroxidase (POD), superoxide dismutase (SOD), soluble protein levels and the PSII maximum photochemical quantum efficiency (F_v_/F_m_) were higher and the hydrogen peroxide (H_2_O_2_) and malondialdehyde (MDA) contents were lower after 4 h of high-temperature treatment than at other time points. Therefore, samples from 0 h (plants kept under normal conditions that were used as the control) and 4 h of treatment were sequenced. Three biological replicates were used for each time point, totaling 12 samples used for sequencing.

RNA-seq was completed by Huada Gene Biological Company (Wuhan, China, *http://www.genomics.cn/*). RNA was extracted from the leaf of the parent, and qualified RNA samples were used to construct the cDNA library. The sequencing of each cDNA library was carried out on the BGISEQ-500 platform. Sequences of low quality, with joint contamination or with a high unknown base (N) content were filtered by SOAPnuke software from the raw reads obtained after sequencing [73]. All filtered clean reads were mapped to the *SL2.50* (*https://www.ncbi.nlm.nih.gov/assembly/GCF_000188115.3*) tomato reference genome using HISAT; then, new transcript prediction, SNP and InDel identification and differential splicing for gene detection were performed [74]. The new transcripts with the potential to encode proteins were added to the reference sequence to form a complete reference sequence, and the gene expression level of each sample was calculated using RSEM [75]. Finally, the differentially expressed genes among different samples were detected as those with a fold change ≥2 or ≤ − 2 and a *P*-value ≤0.001 [76], and the DEGs were further analyzed by the Gene Ontology (GO) and Kyoto Encyclopedia of Genes and Genomes (KEGG) databases to assess their functional enrichment.

### Real-time quantitative PCR

The quality of the RNA-seq results was assessed using qRT-PCR of triplicate samples used for the RNA-seq experiment, and further screening of candidate genes was carried out with samples treated with high temperature for 0, 4, 8 or 12 h. All experiments involved 3 biological replicates and 3 technical replicates. TRIzol reagent (Invitrogen, CA, USA) was used to extract total RNA, and ABI (Shanghai, China) SYBR® Select Master Mix (2 x) kits and an Eppendorf real-time PCR machine (Hamburg, Germany) were used for qRT-PCR analysis.

## Additional files

Additional file 1: **Table S1.** Statistics of linkage map developed in this study. (DOCX 16 kb)

Additional file 2: **Table S2.** The statistics and evaluation of sequencing data generated from the extreme pools and parents in QTL-seq. (DOCX 16 kb)

Additional file 3: **Table S3.** The comparison results of the extreme pools and parents with the reference *SL 2.50* in QTL-seq. (DOCX 15 kb)

Additional file 4: **Table S4.** The statistics of sequencing data and genome comparison with the reference *SL 2.50* in RNA-seq. (DOCX 16 kb)

Additional file 5: **Table S5.** Summary of RNA-seq. (DOCX 15 kb)

Additional file 6: **Table S6.** The public DEGs of parents in RNA-seq. (XLSX 974 kb)

Additional file 7: **Table S7.** The specific DEGs of LA1698 in RNA-seq. (XLSX 271 kb)

Additional file 8: **Table S8.** The specific DEGs of LA2093 in RNA-seq. (XLSX 319 kb) (XLSX 319 kb)

Additional file 9: **Table S9.** The DEGs detected in major QTLs. (XLSX 41.6 kb) (XLSX 44 kb)

Additional file 10: **Table S10.** Primers used to real-time qRT-PCR. (DOCX 17 kb)

Additional file 11: **Table S11.** GO and KEGG analysis of 12 candidates genes selected by RNA-seq and qRT-PCR. (XLSX 11.0 kb) (XLSX 11 kb)

Additional file 12: **Figure S1**. The frequency distribution of the three heat tolerance-related physiological indexes in the F_2_ population. (DOCX 685 kb)

Additional file 13: **Figure S2**. Genetic linkage map of tomato and positions of QTLs for heat tolerance. (DOCX 476 kb)

Additional file 14: **Figure S3**. Frequency distribution of heat injury index in the F_2_ population. (DOCX 71 kb)

Additional file 15: **Figure S4**. The comparison of the relative expression measured by qRT-PCR and RNA-seq. (DOCX 284 kb)

Additional file 16: **Figure S5**. GO (a) and KEGG (b) classification of the DEGs. (DOCX 92 kb)

Additional file 17: **Figure S6**. Relative expression analysis of 25 candidate genes in 4, 8 and 12 h of the parents. (DOCX 207 kb)

Additional file 18: **Figure S7**. The screening of time-point for RNA-seq. (DOCX 408 kb)

## Data Availability

All data generated or analysed during this study are included in this published article and its supplementary information files. All RNA sequencing data from the present study have been submitted to the NCBI sequence read archive (SRA) under accession numbers: SAMN12591987 (*https://www.ncbi.nlm.nih.gov/biosample/12591987*), SAMN12591988 (*https://www.ncbi.nlm.nih.gov/biosample/12591988*), SAMN12591989 (*https://www.ncbi.nlm.nih.gov/biosample/12591989*), and SAMN12591990 (*https://www.ncbi.nlm.nih.gov/biosample/12591990*).

## Abbreviations

APX: Ascorbic acid reductase
CC: Chlorophyll content
DEGs: Differentially expressed genes
F_v_/F_m_: Maximum photochemical quantum efficiency
GO: Gene ontology
GR: Glutathione reductase
H_2_O_2_: Hydrogen peroxide
HII: Heat injury index
HSB: Heat-sensitive bulk
HSR: high-temperature stress-responsive
HTB: Heat-tolerant bulk
ICIM: Inclusive composite interval mapping
KEGG: Kyoto Encyclopedia of Genes and Genomes
LG: Linkage group
MAPK: Mitogen-activated protein kinases
MDA: Malondialdehyde
NGS: Next-generation sequencing
NO: Nitric oxide
POD: Peroxidase
PSII: Photosystem II
qRT-PCR: Real-time quantitative PCR
QTL: Quantitative trait locus
QTL-seq: Quantitative trait locus sequence
REC: Relative electrical conductivity
RNA-seq: RNA sequence
RT: Reverse transcriptase
SlARG1: Arginase 1
SlCathB2: Cathepsin B-like protease 2
SlGST: Glutathione S-transferase zeta class-like isoform X1
SlUBC5: Ubiquitin-conjugating enzyme E2-23 kDa
SOD: Superoxide dismutase
TGRC: Tomato Genetic Resource Center
