# Supplementary material for: Identification of heat-tolerance QTLs and high-temperature stress-responsive genes through conventional QTL mapping, QTL-seq and RNA-seq in tomato
Source: BMC Plant Biol. 2019 Sep 11;19:398. doi: 10.1186/s12870-019-2008-3 (PMC6739936; doi:10.1186/s12870-019-2008-3)
Supplement: Supplementary file 1 — Table S1. Statistics of linkage map developed in this study. (DOCX 16 kb) [file 12870_2019_2008_MOESM1_ESM.docx]

**Additional file 1: Table S1** Statistics of linkage map developed in this study

| Chr. | Map length (cM) | No. of loci | Average marker interval (cM) |
| --- | --- | --- | --- |
| chr1 | 169.37 | 10 | 16.94 |
| chr2 | 102.41 | 10 | 10.24 |
| chr3 | 148.14 | 15 | 9.88 |
| chr4 | 144.32 | 11 | 13.12 |
| chr5 | 109.30 | 9 | 12.14 |
| chr6 | 127.85 | 12 | 10.65 |
| chr7 | 118.31 | 14 | 8.45 |
| chr8 | 163.88 | 16 | 11.49 |
| chr9 | 106.43 | 7 | 15.20 |
| chr10 | 101.31 | 15 | 6.75 |
| chr11 | 69.89 | 11 | 6.35 |
| chr12 | 142.61 | 7 | 20.37 |
| SUM/mean | 1503.82 | 137 | 10.98 |
