# Supplementary material for: Identification of heat-tolerance QTLs and high-temperature stress-responsive genes through conventional QTL mapping, QTL-seq and RNA-seq in tomato
Source: BMC Plant Biol. 2019 Sep 11;19:398. doi: 10.1186/s12870-019-2008-3 (PMC6739936; doi:10.1186/s12870-019-2008-3)
Supplement: Supplementary file 2 — Table S2. The statistics and evaluation of sequencing data generated from the extreme pools and parents in QTL-seq. (DOCX 16 kb) [file 12870_2019_2008_MOESM2_ESM.docx]

**Additional file 2: Table S2** The statistics and evaluation of sequencing data generated from the extreme pools and parents in QTL-seq

| Sample ID | Clean Reads | Clean Base(bp) | GC(%) | Q30(%) | SNP Number |
| --- | --- | --- | --- | --- | --- |
| S-Pool | 85,307,408 | 25,047,615,247 | 35.50 | 95.67 | 5,836,540 |
| T-Pool | 83,673,715 | 24,569,062,278 | 35.39 | 95.62 | 5,840,665 |
| LA1698 | 48,383,616 | 14,167,494,142 | 35.45 | 95.41 | 4,491,207 |
| LA2093 | 42,900,716 | 12,586,800,636 | 35.08 | 95.56 | 3,521,275 |
