# Supplementary material for: Identification of heat-tolerance QTLs and high-temperature stress-responsive genes through conventional QTL mapping, QTL-seq and RNA-seq in tomato
Source: BMC Plant Biol. 2019 Sep 11;19:398. doi: 10.1186/s12870-019-2008-3 (PMC6739936; doi:10.1186/s12870-019-2008-3)
Supplement: Supplementary file 3 — Table S3. The comparison results of the extreme pools and parents with the reference SL 2.50 in QTL-seq. (DOCX 15 kb) [file 12870_2019_2008_MOESM3_ESM.docx]

**Additional file 3: Table S3** The comparison results of the extreme pools and parents with the reference *SL 2.50* in QTL-seq.

| Sample ID | Mapped Ratio(%) | Coverage Bases (bp) | Coverage 1X(%) | Average Depth(X) |
| --- | --- | --- | --- | --- |
| S-Pool | 99.49 | 736,981,497 | 89.46 | 25.11 |
| T-Pool | 99.49 | 736,984,682 | 89.46 | 24.55 |
| LA1698 | 99.54 | 729,856,704 | 88.60 | 14.50 |
| LA2093 | 99.37 | 718,223,165 | 87.19 | 12.81 |
