# Supplementary material for: Identification of heat-tolerance QTLs and high-temperature stress-responsive genes through conventional QTL mapping, QTL-seq and RNA-seq in tomato
Source: BMC Plant Biol. 2019 Sep 11;19:398. doi: 10.1186/s12870-019-2008-3 (PMC6739936; doi:10.1186/s12870-019-2008-3)
Supplement: Supplementary file 4 — Table S4. The statistics of sequencing data and genome comparison with the reference SL 2.50 in RNA-seq. (DOCX 16 kb) [file 12870_2019_2008_MOESM4_ESM.docx]

**Additional file 4: Table S4** The statistics of sequencing data and genome comparison with the reference *SL 2.50* in RNA-seq

| Sample | Total Raw Reads (M) | Total Clean Reads (M) | Total Clean Bases(Gb) | Clean Reads Q20(%) | Clean Reads Q30(%) | Total Mapping(%) |
| --- | --- | --- | --- | --- | --- | --- |
| LA1698_0_1 | 72.22 | 69.5 | 6.95 | 98.69 | 92.96 | 94.16 |
| LA1698_0_2 | 72.22 | 68.52 | 6.85 | 97.58 | 89.85 | 94.25 |
| LA1698_0_3 | 77.2 | 72.76 | 7.28 | 97.64 | 90 | 94.16 |
| LA1698_4_1 | 74.71 | 70.8 | 7.08 | 97.54 | 89.65 | 95.49 |
| LA1698_4_2 | 72.22 | 69.73 | 6.97 | 98.74 | 93.12 | 94.5 |
| LA1698_4_3 | 69.73 | 67.16 | 6.72 | 98.69 | 92.83 | 94.89 |
| LA2093_0_1 | 74.71 | 70.66 | 7.07 | 97.64 | 90.05 | 92.65 |
| LA2093_0_2 | 77.2 | 71.77 | 7.18 | 97.64 | 90.01 | 92.4 |
| LA2093_0_3 | 69.73 | 66.17 | 6.62 | 97.9 | 90.43 | 93.47 |
| LA2093_4_1 | 71.77 | 68.18 | 6.82 | 97.59 | 89.82 | 92.95 |
| LA2093_4_2 | 71.02 | 66.28 | 6.63 | 97.51 | 89.61 | 92.3 |
| LA2093_4_3 | 53.68 | 52.67 | 5.27 | 97.94 | 90.81 | 92.71 |
