# Supplementary material for: Identification of heat-tolerance QTLs and high-temperature stress-responsive genes through conventional QTL mapping, QTL-seq and RNA-seq in tomato
Source: BMC Plant Biol. 2019 Sep 11;19:398. doi: 10.1186/s12870-019-2008-3 (PMC6739936; doi:10.1186/s12870-019-2008-3)
Supplement: Supplementary file 5 — Table S5. Summary of RNA-seq. (DOCX 15 kb) [file 12870_2019_2008_MOESM5_ESM.docx]

**Additional file 5: Table S5** Summary of RNA-seq

| Expression gene | Known gene | Novel gene | Total novel transcript | Novel isoform | Novel protein-coding transcript | Noncoding transcript |
| --- | --- | --- | --- | --- | --- | --- |
| 23,458 | 22,612 | 846 | 14,639 | 11,739 | 857 | 2077 |
