# Supplementary material for: Identification of heat-tolerance QTLs and high-temperature stress-responsive genes through conventional QTL mapping, QTL-seq and RNA-seq in tomato
Source: BMC Plant Biol. 2019 Sep 11;19:398. doi: 10.1186/s12870-019-2008-3 (PMC6739936; doi:10.1186/s12870-019-2008-3)
Supplement: Supplementary file 10 — Table S10. Primers used to real-time qRT-PCR. (DOCX 17 kb) [file 12870_2019_2008_MOESM10_ESM.docx]

| Gene ID | Forward primer sequence（5'-3'） | Reverse primer sequence（5'-3'） | Product length (bp) |
| --- | --- | --- | --- |
| *actin* | GTGAAAGAAAAGCTCGCTTACA | GCTCATAGCTCTTCTCAACAGA | 91 |
| 101055518 | AGCCGCTTACTAGTGAAGAATT | GCCATAGCTTTAAGAAAGACCG | 115 |
| 101244831 | GGTTCTTAACTTGGATTCGTCG | GGATCTTCCTCTTAGGGTAAGC | 106 |
| 101250361 | TTTGATCGATTCATTGGACGTG | ACCAGCACGAATAGTTAGAACA | 96 |
| 101251441 | CTCTTGTTTTTGTCTCACCCTG | CTTGTTGTACCATTCTCACCAC | 199 |
| 101251744 | AAAGAGTTTGATGCACGAAAGG | TGTCGAAGTAAGGATCACACTC | 280 |
| 108281121 | TGCGTTCAAACTTAGACAACAG | CTTTATCCGAATTTGAGACGGG | 188 |
| 101266182 | AGACCGTGTTAAGTACTTAGGC | AATAGCTCAGGGAAGACACATC | 198 |
| 101249959 | ACTATTTGGACCAAGACCAGAG | CTCTCCCAATTCAGTTAGTCGT | 135 |
| 101252525 | GGAGCTCTTGAAAAATTGCTGA | CAATTTGAGGTGCCAAAAACAC | 94 |
| 101254131 | TTTGCAACTCTGGAACATAAGC | ACCTCTTCATCATAGCGAATGT | 162 |
| 101258003 | GCAGATATCATAATCGCTGCTG | CGACATCAATAATTACTGCCCC | 85 |
| 101258305 | CAAGGCAGATAATTTCGTCGTT | TACTATCAATCGTCAGTGCCTC | 173 |
| 101259683 | CAAGGCAAAAACTTCAATGCTG | CCAAGATAAATCAGCTTCTGGC | 210 |
| 101264556 | TTGCAAGATTATCACTTGAGCG | TGATGACCTTATTAATCGCGGA | 216 |
| 101264759 | ATGAGTTCAGAAAAGCAACCAC | AATATTGCTGCATCTGATGGAC | 100 |
| 101265167 | GTGAAACACCCTTTGGAGAATC | CACCTAGAAGGAACAGAACCAA | 151 |
| 101265477 | AGTCGCTAGAAAAAGTACGGAA | AACAATACCCTCTCTGTCAGTG | 82 |
| 101265863 | GTGTGGAAAATAAGGGTGGAAC | TCATCAACATTTGGGTGGTAGA | 95 |
| 543594 | ATGCTTGTTGCTCTTTATTCGG | CAGCAAGCAAACAGTAGAAACT | 224 |
| 543727 | ATCAGCCAATTATCACAACTGC | CCTGGCTGTATGAATAAAAGGC | 83 |
| 543944 | GAGTTCCTCTGGGACATAACTC | AGTTGTAGAGTTTGTACTGCCA | 101 |
| 101254424 | CATGTTCTTCGGTACAACAGTC | GATGTTTTCCCAGCCTTACATG | 138 |
| 101265772 | TGTAGGCAGAGAACTGTTACAG | GATGATTTGCTCGAGTCTTGAC | 137 |
| 100301908 | GCTAAGAGAGAGGTGAATCGTT | TCGTAGCTACAATCATGACGAT | 128 |
| 101264761 | GAAGCTTACGTTGGTAAATCCC | AGAATCATGACCAAAAAGACGC | 162 |
| 101244597 | AACATAAGGTCCTCGAGTTTGT | TACATGGACTAAAGCCAACACT | 165 |
| 101245918 | AAGACATAGAAGTCGATCCACC | GAAAGCCATTGCAACAAAGAAC | 140 |
| 101246759 | TAGCAAAGTGTGAATGTTGTGG | GTCCACATAGCCATTTTCCATC | 96 |
| 101249557 | GCTTAATTTTGACTCACGTTGC | TAGAAGAGGTACTTGACAGCAC | 84 |

**Additional file 10: Table S10** Primers used to real-time qRT-PCR
