# Supplementary material for: Identification of heat-tolerance QTLs and high-temperature stress-responsive genes through conventional QTL mapping, QTL-seq and RNA-seq in tomato
Source: BMC Plant Biol. 2019 Sep 11;19:398. doi: 10.1186/s12870-019-2008-3 (PMC6739936; doi:10.1186/s12870-019-2008-3)
Supplement: Supplementary file 12 — Figure S1. The frequency distribution of the three heat tolerance-related physiological indexes in the F2 population. (DOCX 685 kb) [file 12870_2019_2008_MOESM12_ESM.docx]

**a b**

**c**

**Additional file 12: Figure S1** The frequency distribution of the three heat tolerance-related physiological indexes in the F_2_ population. **(a)**, **(b)** and **(c)** stand for the frequency distribution of REC, F_v_/F_m_ and CC, respectively. REC indicates relative electrical conductivity, F_v_/F_m_ indicates maximum photochemical quantum efficiency, CC indicates chlorophyll content.
