# Supplementary material for: Identification of heat-tolerance QTLs and high-temperature stress-responsive genes through conventional QTL mapping, QTL-seq and RNA-seq in tomato
Source: BMC Plant Biol. 2019 Sep 11;19:398. doi: 10.1186/s12870-019-2008-3 (PMC6739936; doi:10.1186/s12870-019-2008-3)
Supplement: Supplementary file 13 — Figure S2. Genetic linkage map of tomato and positions of QTLs for heat tolerance. (DOCX 476 kb) [file 12870_2019_2008_MOESM13_ESM.docx]

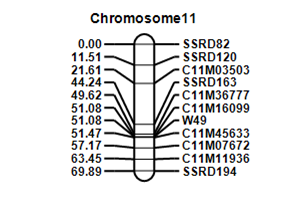

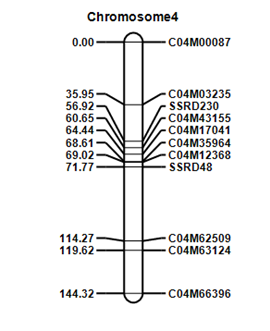

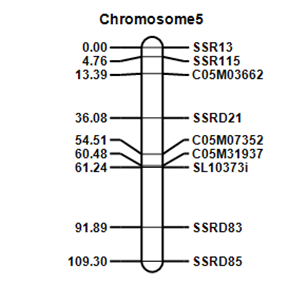

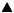

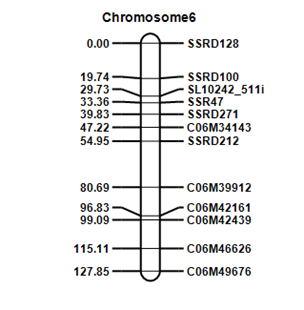

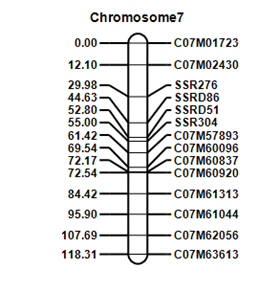

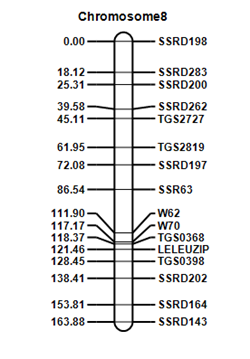

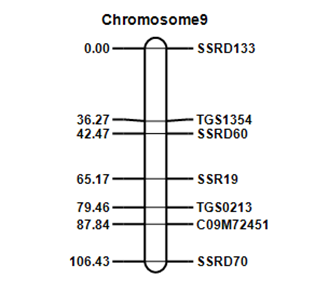

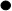

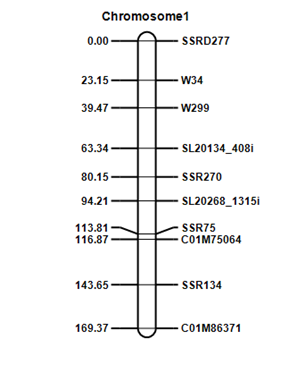

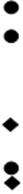

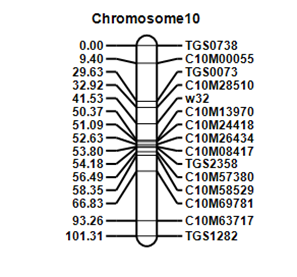

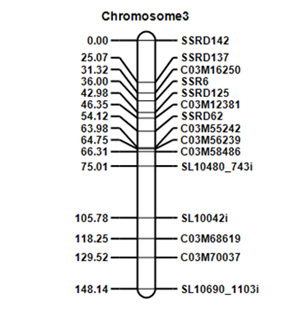

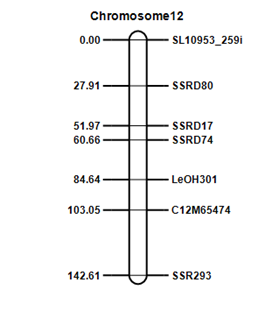

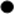

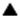

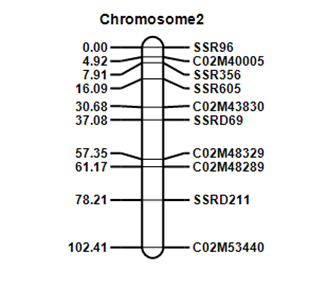


**Additional file 13: Figure S2** Genetic linkage map of tomato and positions of QTLs for heat tolerance. Genetic distances (cM) were shown on the left side of chromosome and marker on the right. represent QTL for REC, CC and F_v_/F_m_, respectively.
