# Supplementary material for: Identification of heat-tolerance QTLs and high-temperature stress-responsive genes through conventional QTL mapping, QTL-seq and RNA-seq in tomato
Source: BMC Plant Biol. 2019 Sep 11;19:398. doi: 10.1186/s12870-019-2008-3 (PMC6739936; doi:10.1186/s12870-019-2008-3)
Supplement: Supplementary file 14 — Figure S3. Frequency distribution of heat injury index in the F2 population. (DOCX 71 kb) [file 12870_2019_2008_MOESM14_ESM.docx]

**
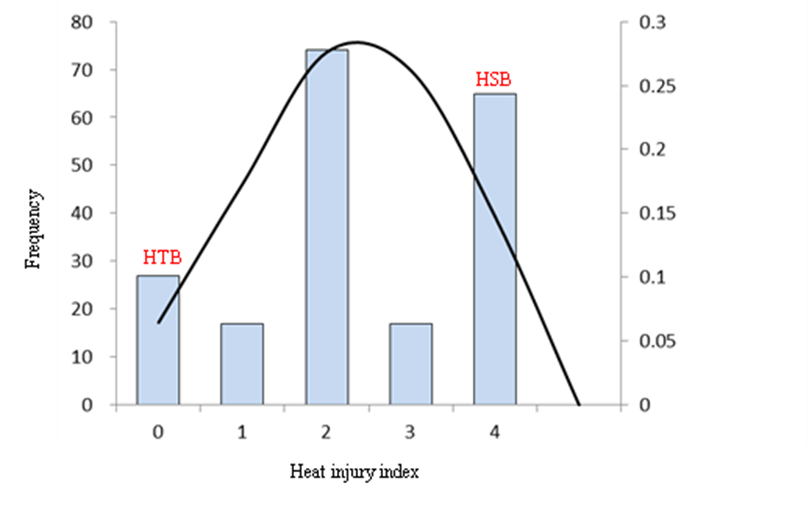
**

**Additional file 14: Figure S3** Frequency distribution of heat injury index in the F_2_ population. The HII of HTB (Heat-tolerant bulk) and HSB (Heat-sensitive bulk) were 0 and 4.
