# Supplementary material for: Identification of heat-tolerance QTLs and high-temperature stress-responsive genes through conventional QTL mapping, QTL-seq and RNA-seq in tomato
Source: BMC Plant Biol. 2019 Sep 11;19:398. doi: 10.1186/s12870-019-2008-3 (PMC6739936; doi:10.1186/s12870-019-2008-3)
Supplement: Supplementary file 15 — Figure S4. The comparison of the relative expression measured by qRT-PCR and RNA-seq. (DOCX 284 kb) [file 12870_2019_2008_MOESM15_ESM.docx]

**a**

**b**

**Additional file 15: Figure S4** The comparison of the relative expression measured by qRT-PCR and RNA-seq. 2 downregulated (*101254424* and *101265772*) and 6 upregulated (*101055518*, *101244831*, *101250361*, *101251441*, *101251744* and *108281121*) heat-tolerant genes within the major QTLs as well as 4 downregulated DEGs (*101244597*, *101245918*, *101246759* and *101249557*) of LA2093 **(a)** and LA1698 **(b)** were measured.
