# Supplementary material for: Identification of heat-tolerance QTLs and high-temperature stress-responsive genes through conventional QTL mapping, QTL-seq and RNA-seq in tomato
Source: BMC Plant Biol. 2019 Sep 11;19:398. doi: 10.1186/s12870-019-2008-3 (PMC6739936; doi:10.1186/s12870-019-2008-3)
Supplement: Supplementary file 16 — Figure S5. GO (a) and KEGG (b) classification of the DEGs. (DOCX 92 kb) [file 12870_2019_2008_MOESM16_ESM.docx]

**a**


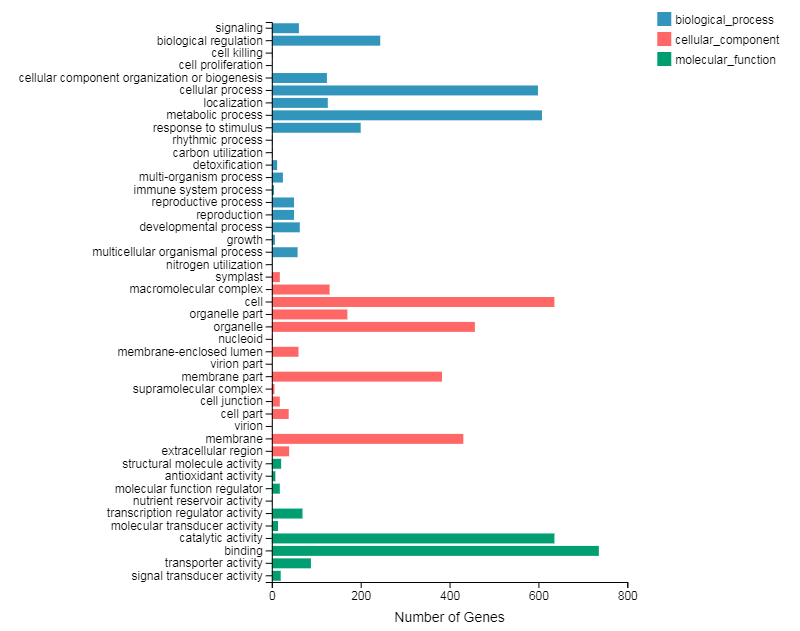


**b**


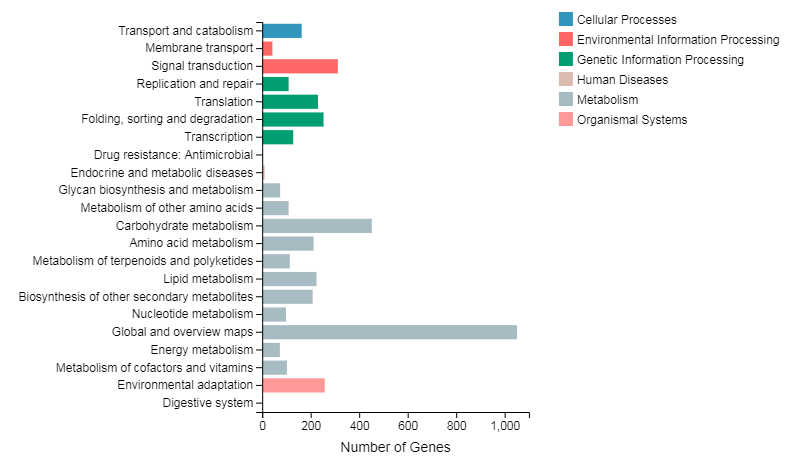


**Additional file 16: Figure S5** GO **(a)** and KEGG **(b)** classification of the DEGs. X-axis represents the number of genes and Y-axis denotes the GO Term or KEGG Pathway.
