# Supplementary material for: Identification of heat-tolerance QTLs and high-temperature stress-responsive genes through conventional QTL mapping, QTL-seq and RNA-seq in tomato
Source: BMC Plant Biol. 2019 Sep 11;19:398. doi: 10.1186/s12870-019-2008-3 (PMC6739936; doi:10.1186/s12870-019-2008-3)
Supplement: Supplementary file 17 — Figure S6. Relative expression analysis of 25 candidate genes in 4, 8 and 12 h of the parents. (DOCX 207 kb) [file 12870_2019_2008_MOESM17_ESM.docx]

**
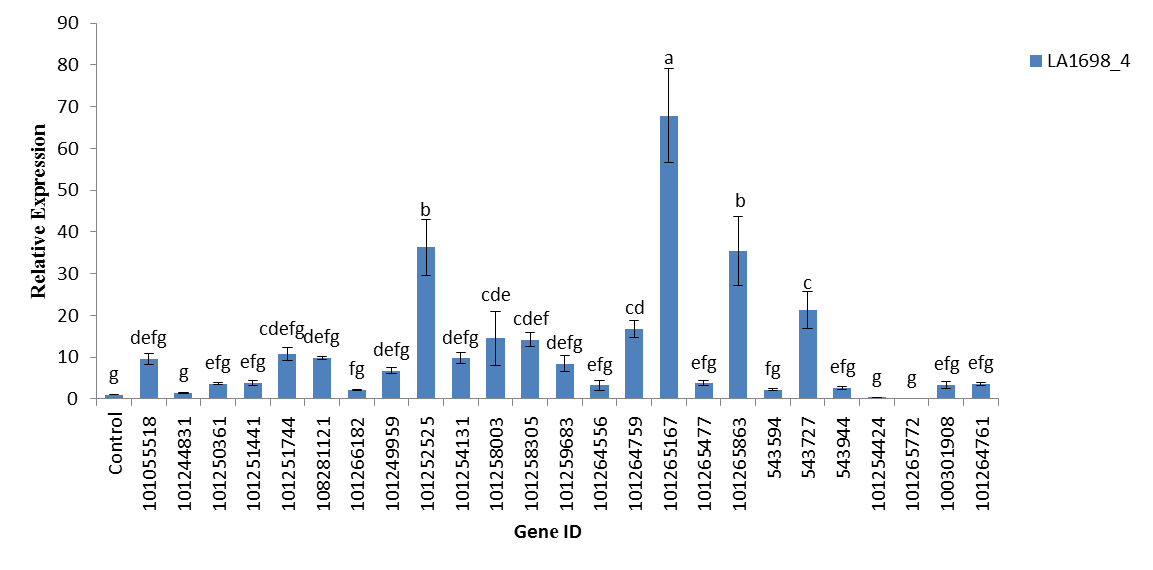
a**

**b**


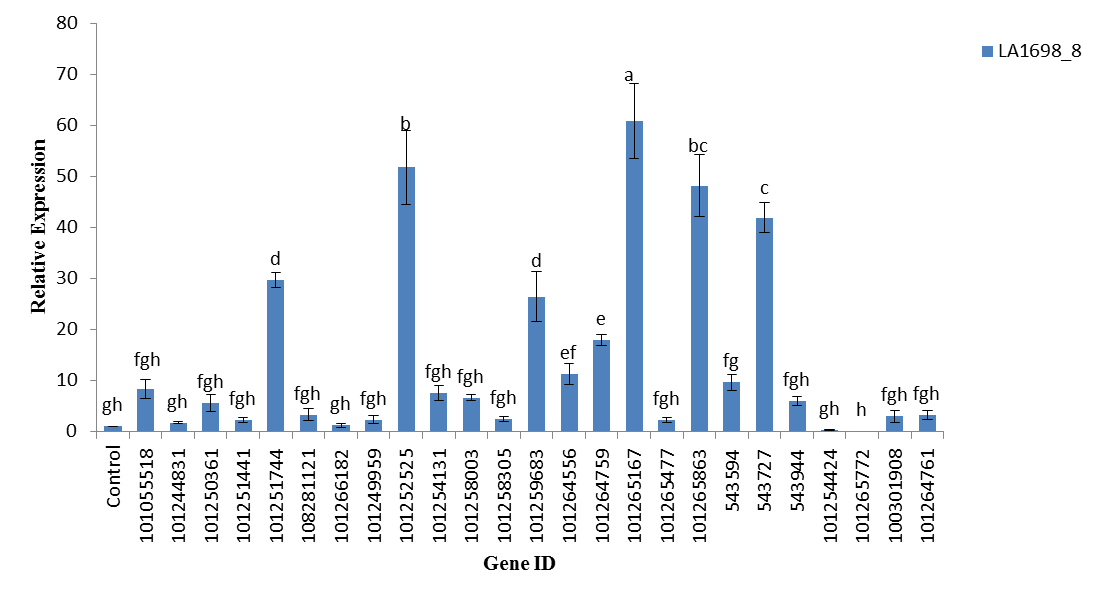


**c**


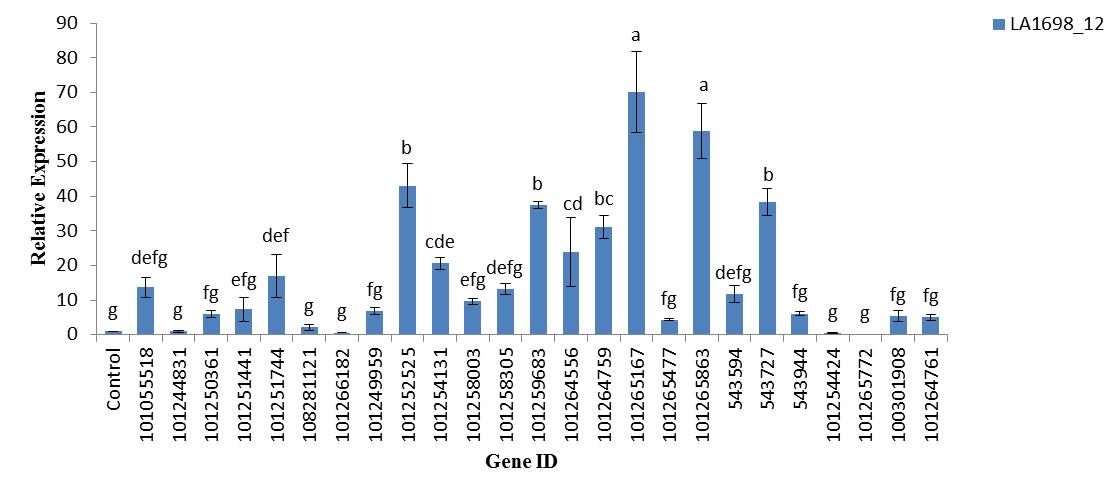


**d**


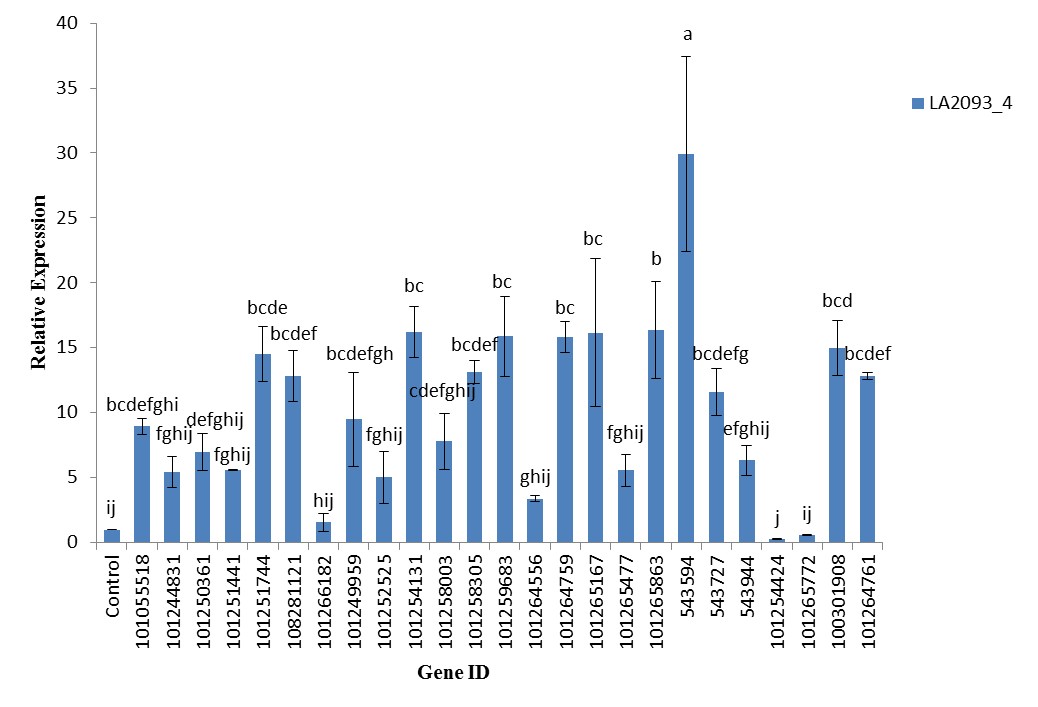


**e**

**
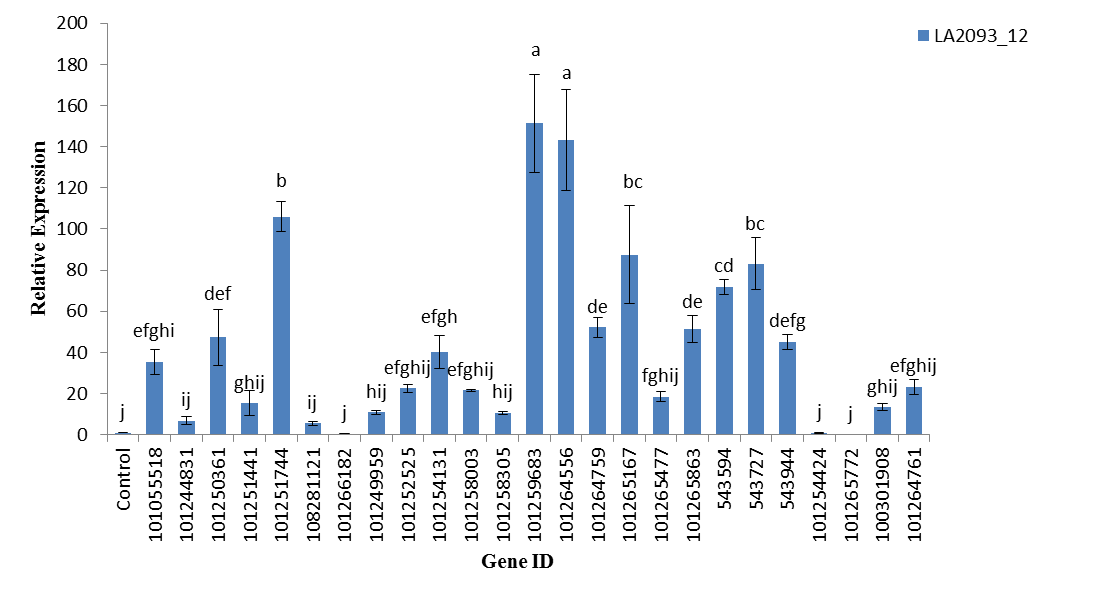

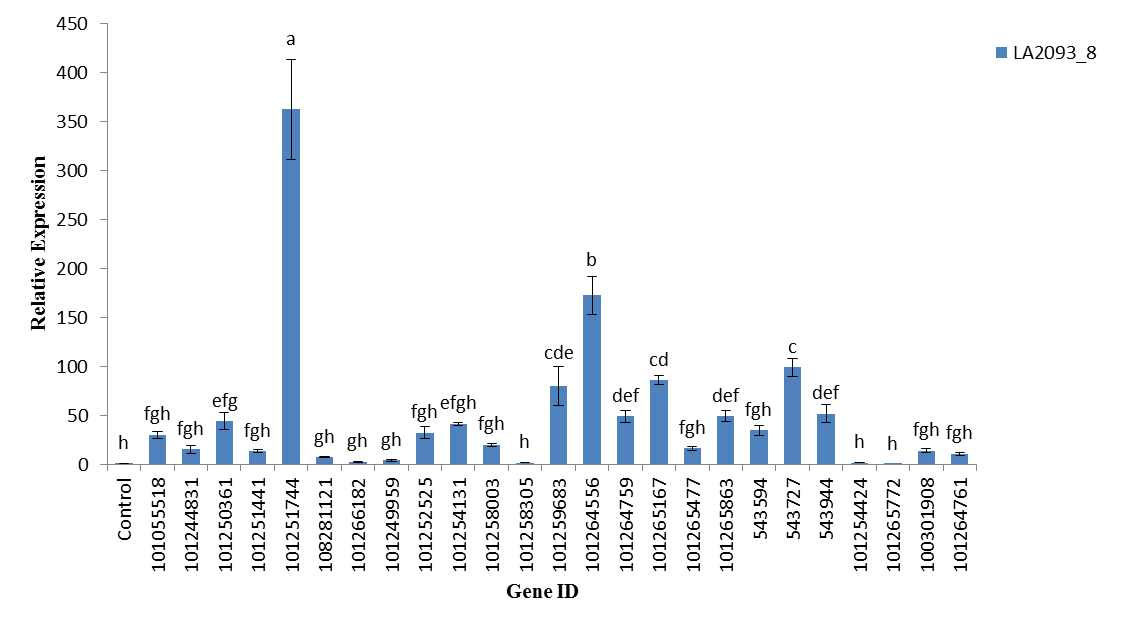
f**

**Additional file 17: Figure S6** Relative expression analysis of 25 candidate genes in 4, 8 and 12h of the parents. **(a)**, **(b)** and **(c)** represent relative expression of 25 candidate genes in each time-point (4, 8 and 12h) of LA1698, respectively. **(d)**, **(e)** and **(f)** represent relative expression of 25 candidate genes in each time-point (4, 8 and 12h) of LA2093, respectively. Error bars represent standard deviations for three replicates. Difference significance test of statistics using an ANOVA.
