# Supplementary material for: Identification of heat-tolerance QTLs and high-temperature stress-responsive genes through conventional QTL mapping, QTL-seq and RNA-seq in tomato
Source: BMC Plant Biol. 2019 Sep 11;19:398. doi: 10.1186/s12870-019-2008-3 (PMC6739936; doi:10.1186/s12870-019-2008-3)
Supplement: Supplementary file 18 — Figure S7. The screening of time-point for RNA-seq. (DOCX 408 kb) [file 12870_2019_2008_MOESM18_ESM.docx]

**a**

**
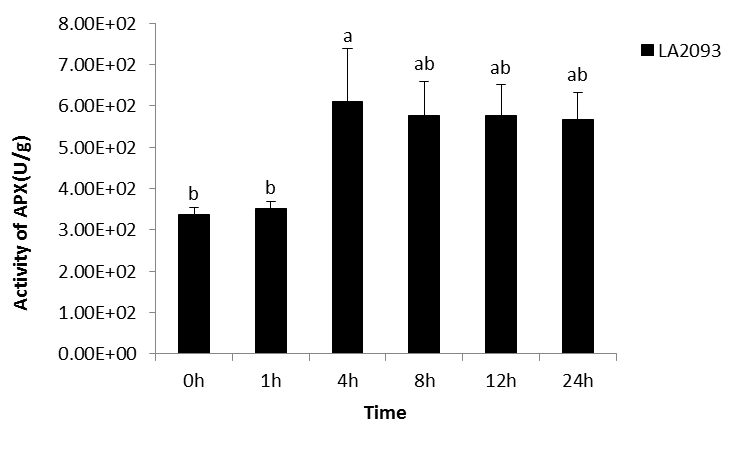

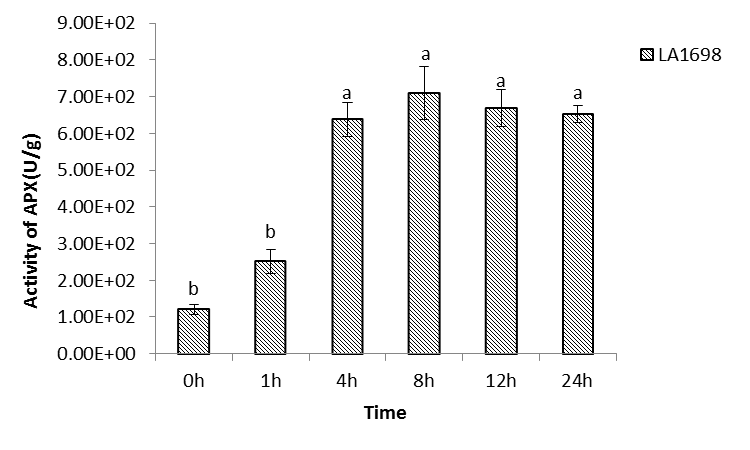
**

**
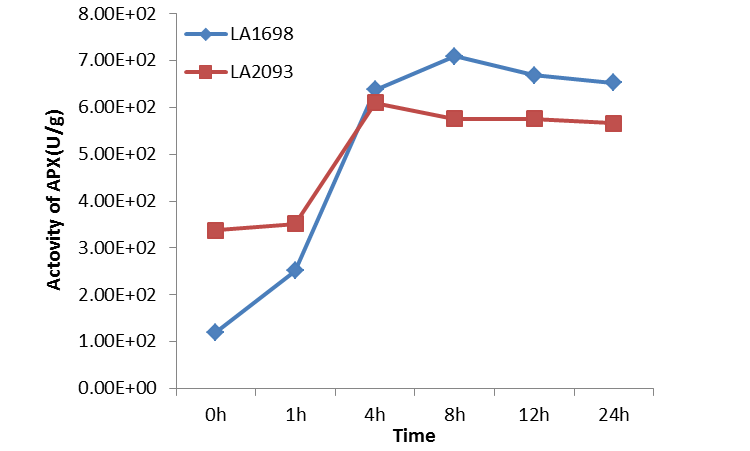
**

**
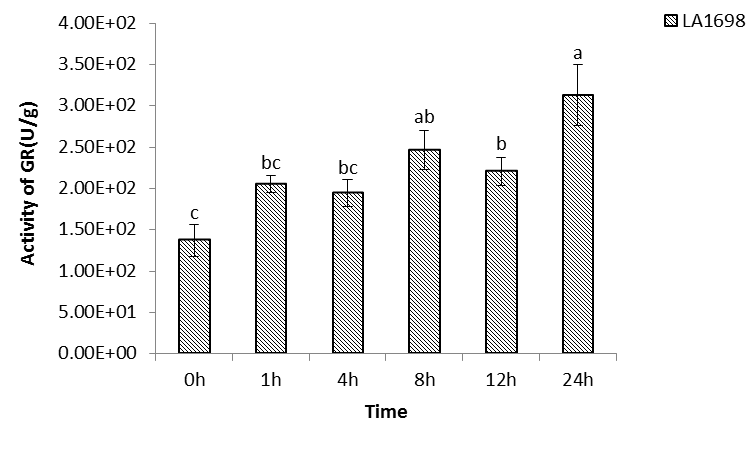

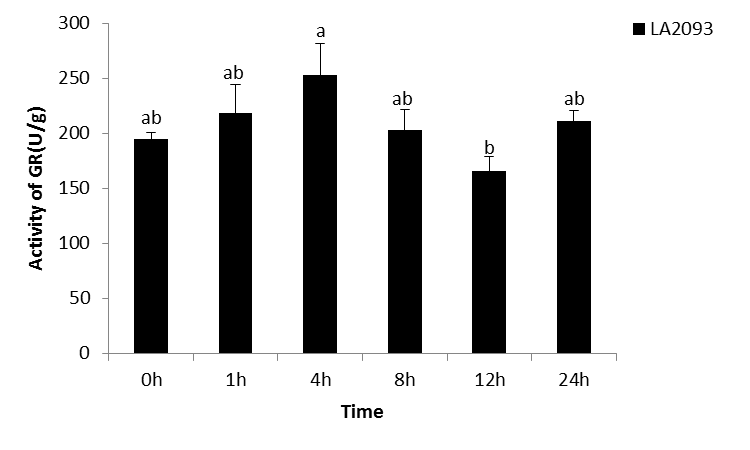
b**

**
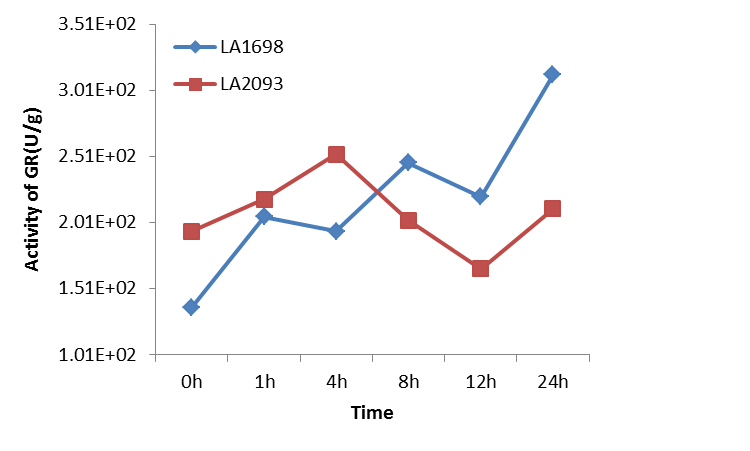
**

**c**

**
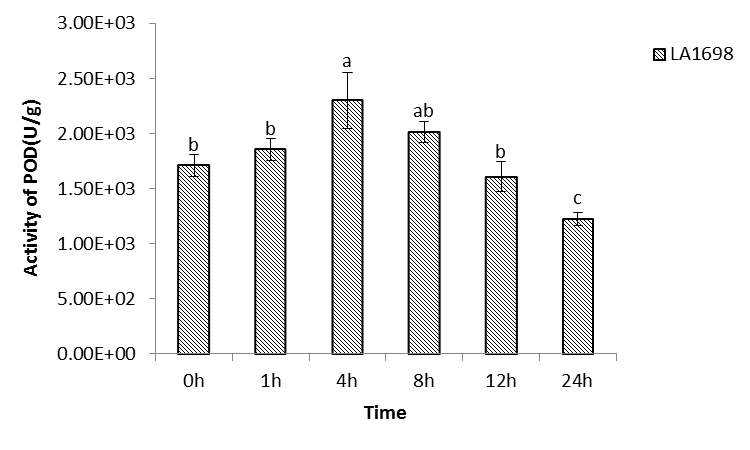

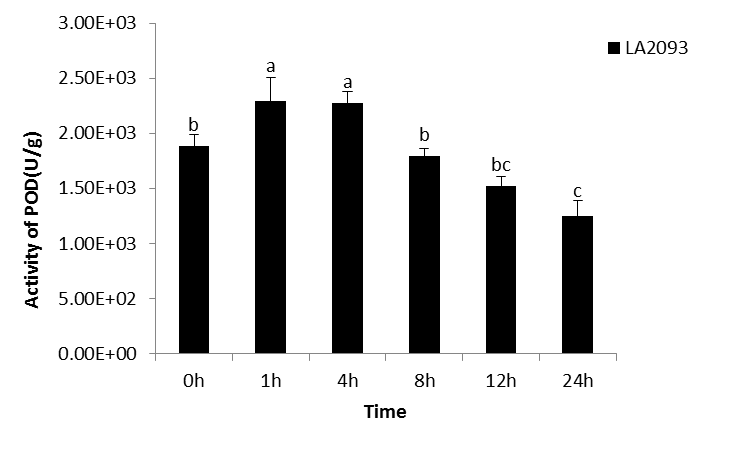
**

**
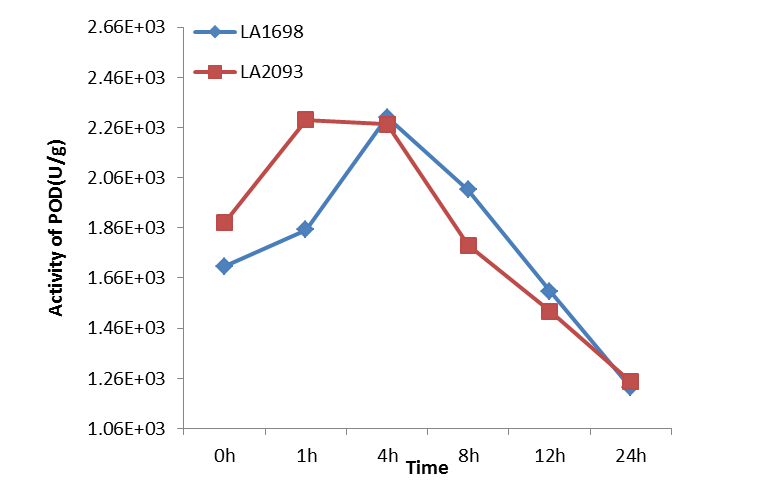
**

**d**

**
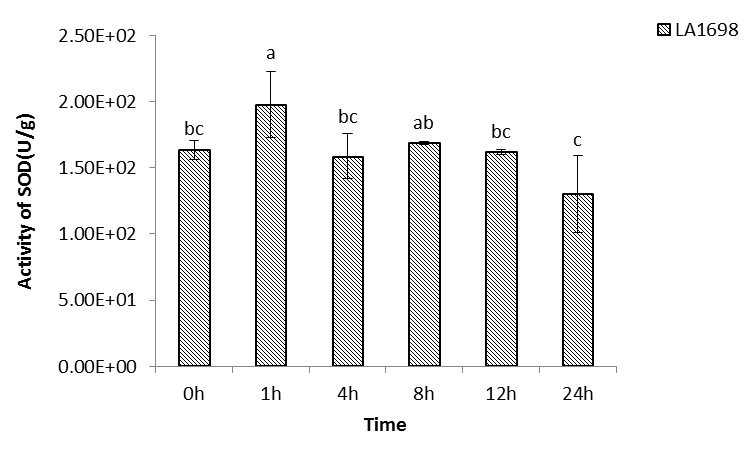

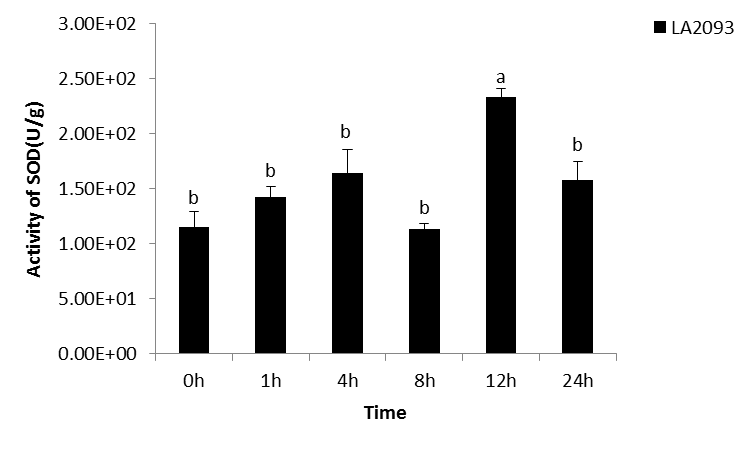
**

**
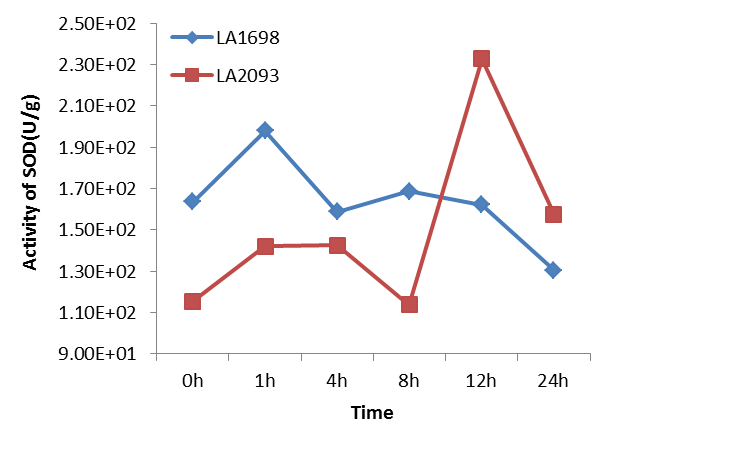
**

**
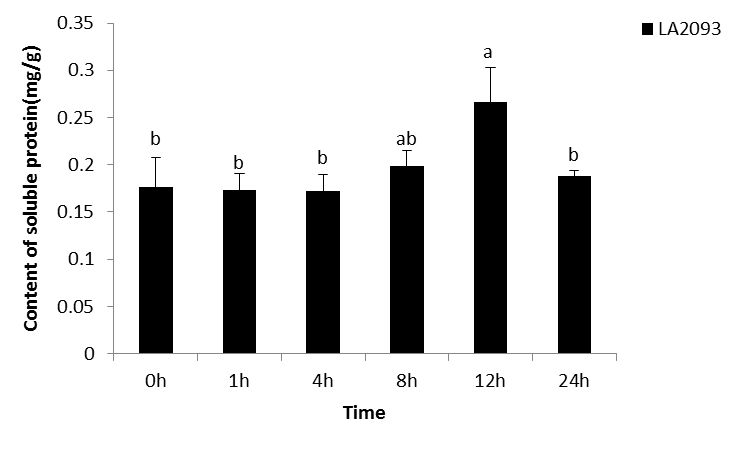

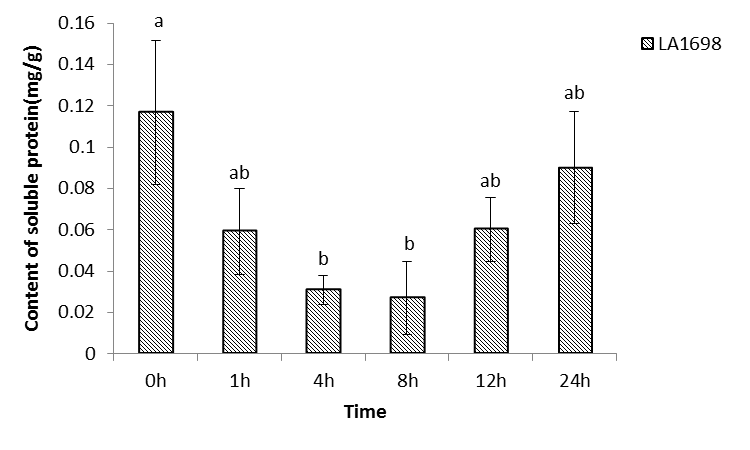
e**


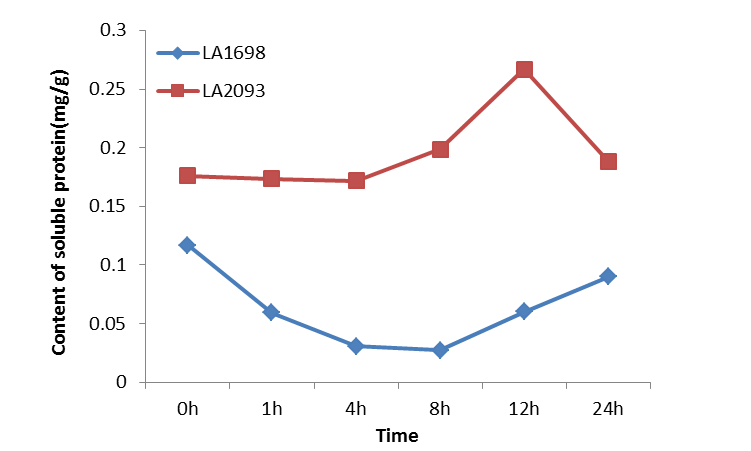


**f**

**
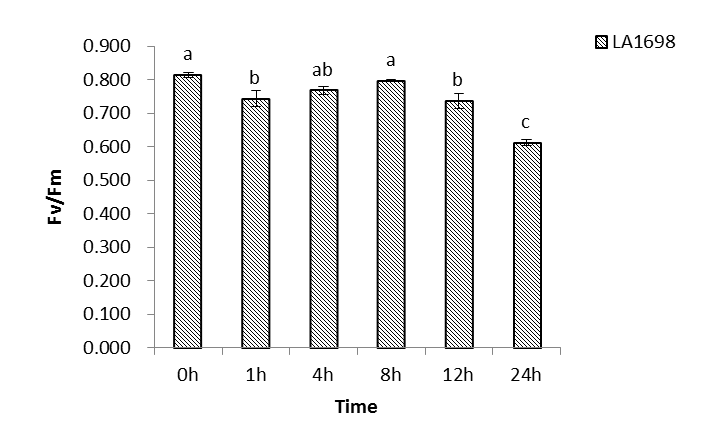

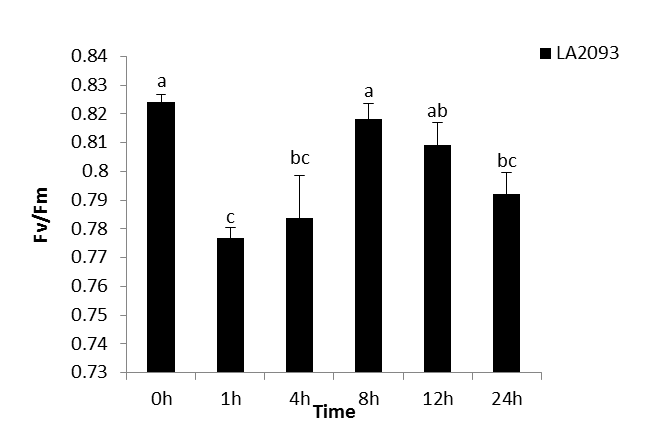
**

**
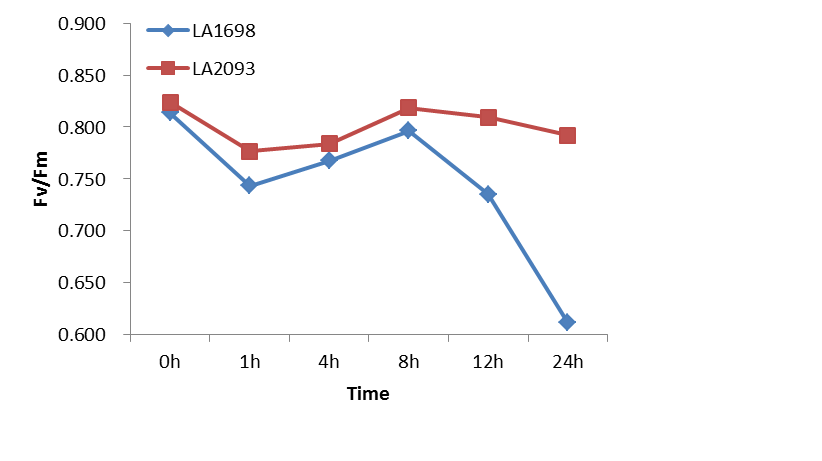
**

**
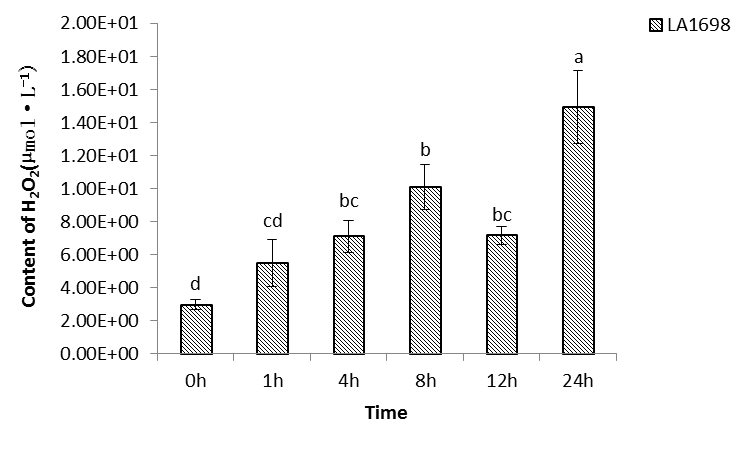
g**

**
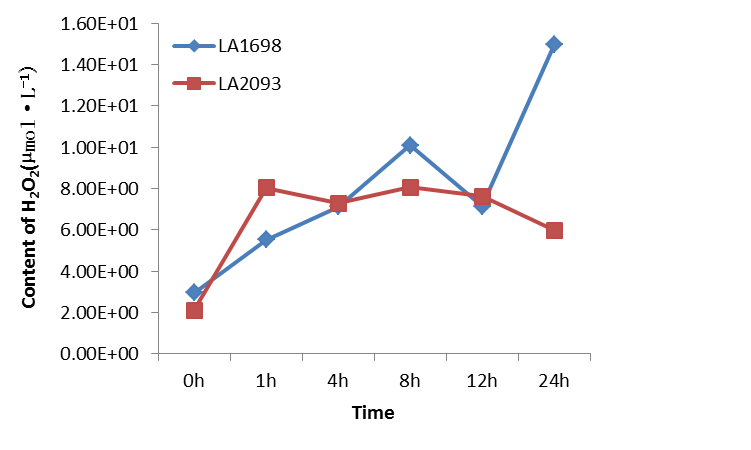

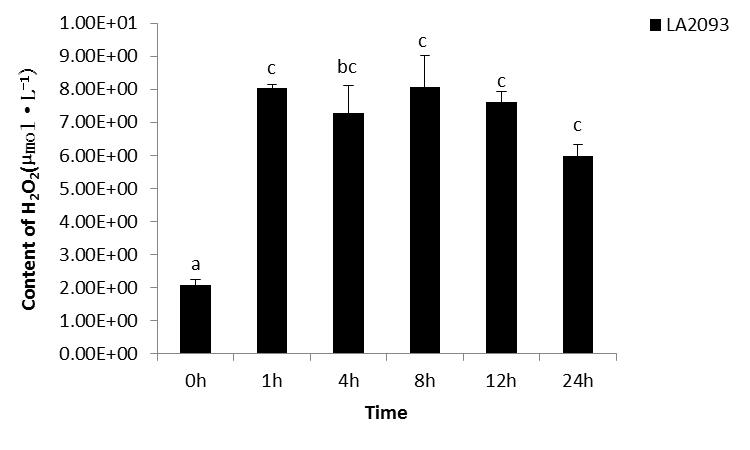
**

**
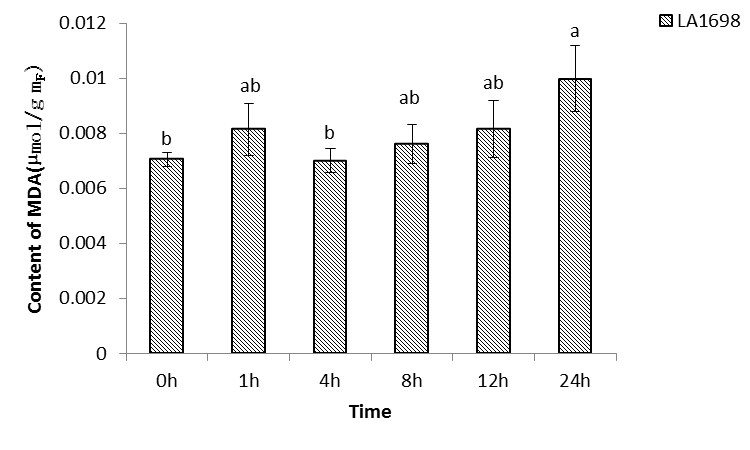
h**

**
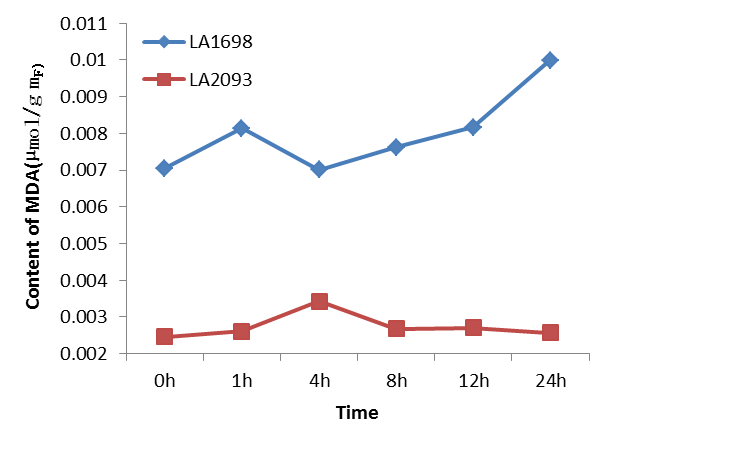

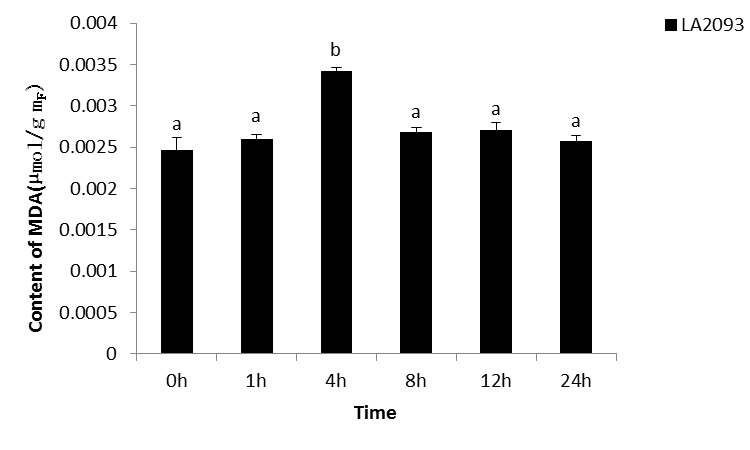
**

**Additional file 18: Figure S7** The screening of time-point for RNA-seq. The time-point was screened by measuring the heat tolerance related physiological traits at 0, 1, 4, 8, 12 and 24h after 40°C high-temperature treatment. Error bars represent standard deviations for three replicates. The red line showed the trend of LA2093, and blue line denoted the trend of LA1698. **(a)** activity of APX; **(b)** activity of GR; **(c)** activity of POD; **(d)** activity of SOD; **(e)** content of soluble protein; **(f)** F_v_/F_m_; **(g)** Content of H_2_O_2_ (µmol·L-1); **(h)** Content of MDA (µmol/g mF). Difference significance test of statistics using an ANOVA.
